# Supplementary material for: WASP Restricts Active Rac to Maintain Cells’ Front-Rear Polarization
Source: Curr Biol. 2019 Dec 16;29(24):4169–4182.e4. doi: 10.1016/j.cub.2019.10.036 (PMC6926487; doi:10.1016/j.cub.2019.10.036)
Supplement: Document S1. Figures S1–S4 [file mmc1.pdf]

Current Biology, Volume *29*

Supplemental Information

WASP Restricts Active Rac to Maintain Cells' Front-Rear Polarization

Clelia Amato, Peter A. Thomason, Andrew J. Davidson, Karthic Swaminathan, Shehab Ismail, Laura M. Machesky, and Robert H. Insall

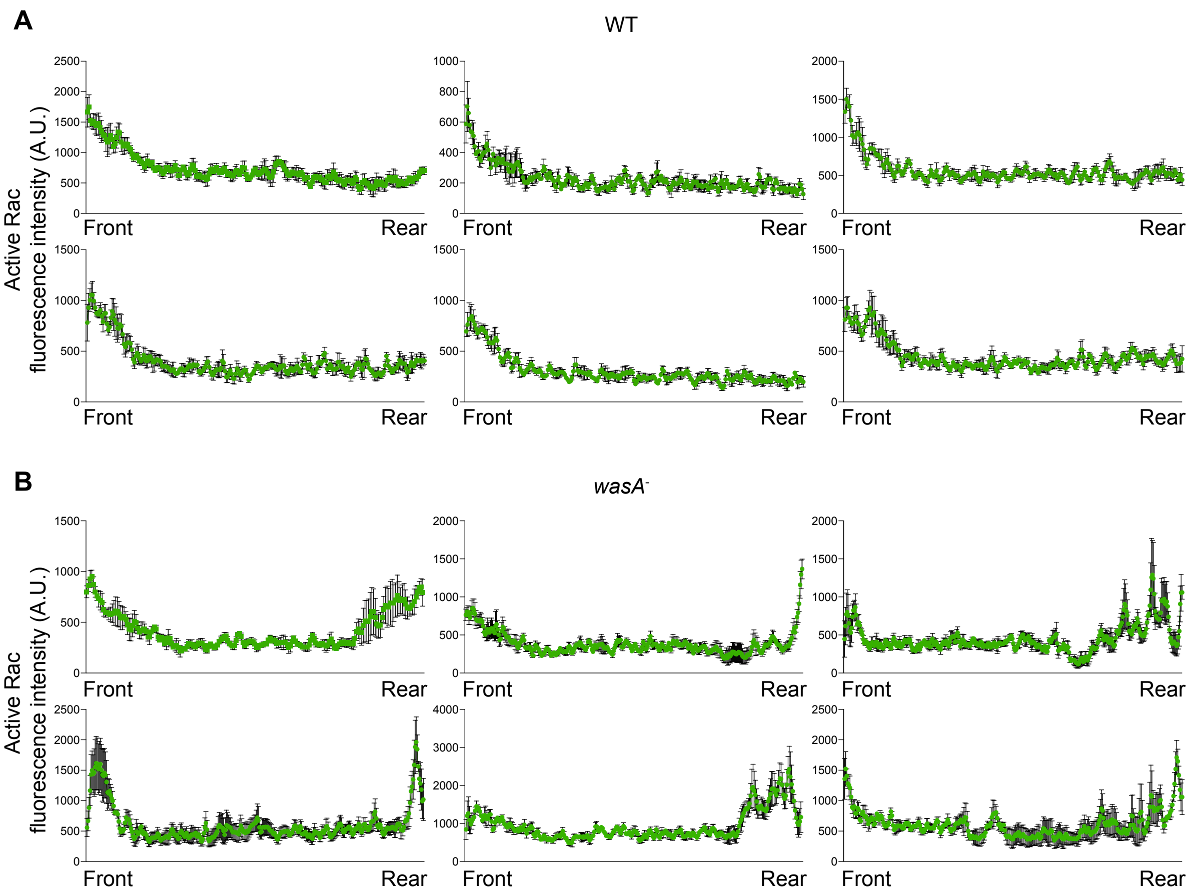
A
WT
Active Rac
fluorescence intensity (A.U.)
Front
Rear
B
wasA-
Active Rac
fluorescence intensity (A.U.)
Front
Rear

Figure S1. *wasA*⁻ cells accumulate active Rac at the rear. Related to Figure 1.

Each plot reports the fluorescence intensity of active Rac marker (PakB CRIB-GFP) along a line connecting front to rear of six representative wild type (WT) (A) and *wasA*⁻ (B) cells at four time-points. Active Rac is accumulated exclusively at the front in WT cells, but both at front and rear in *wasA*⁻ cells.

A

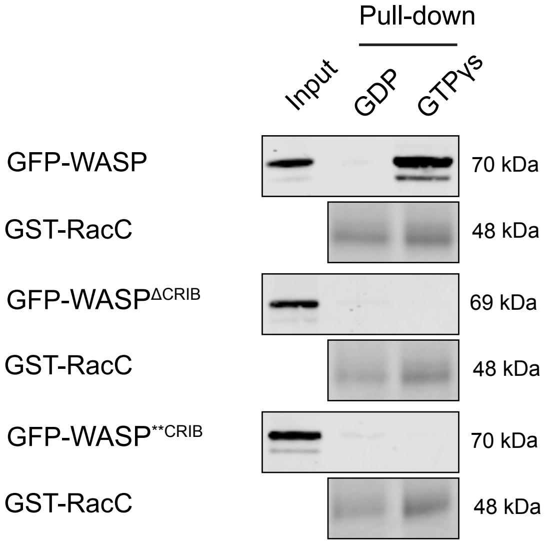
Pull-down
Input
GDP
GTPγs
GFP-WASP
70 kDa
GST-RacC
48 kDa
GFP-WASP$^{\Delta CRIB}$
69 kDa
GST-RacC
48 kDa
GFP-WASP^{**CRIB}
70 kDa
GST-RacC
48 kDa

Figure S2. Mutations in the WASP CRIB motif abrogate binding to active RacC. Related to Figure 2.

(A) GFP-WASP (first panel) interacts with active (GTPγs-bound) RacC, but not with inactive (GDP-bound) RacC. GFP- WASP$^{\Delta CRIB}$ and GFP-WASP^{**CRIB} (third and fifth panels) do not bind RacC, irrespective of its activation state (IB= anti-GFP). Anti-GST immunoblot was performed (second, fourth and sixth panels) to verify the expression of GST-RacC.

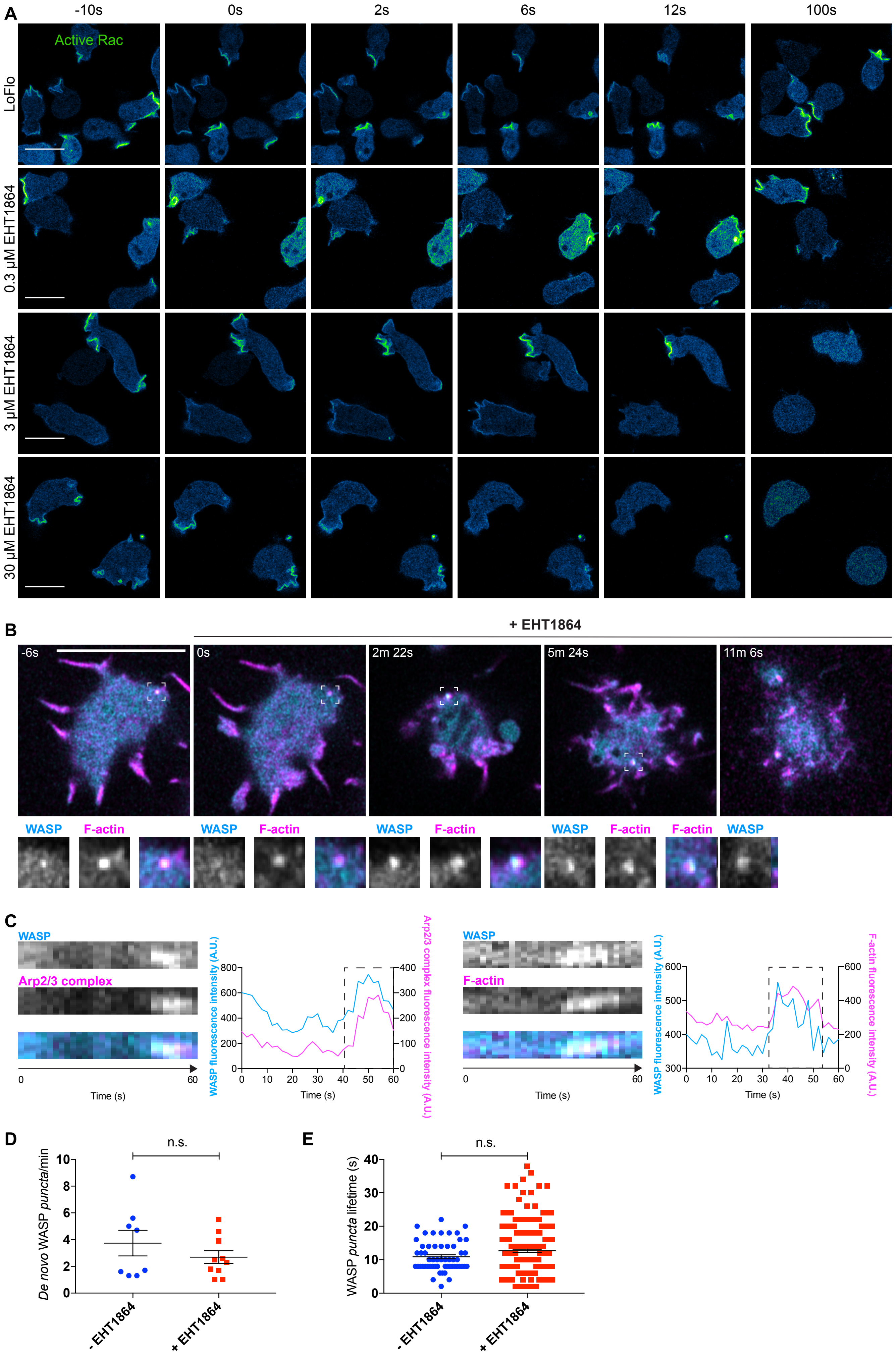

A
-10s
0s
2s
6s
12s
100s
Active Rac
LoFlo
0.3 µM EHT1864
3 µM EHT1864
30 µM EHT1864
B
+ EHT1864
-6s
0s
2m 22s
5m 24s
11m 6s
WASP
F-actin
C
WASP
Arp2/3 complex
Time (s)
WASP fluorescence intensity (A.U.)
Arp2/3 complex fluorescence intensity (A.U.)
F-actin
F-actin fluorescence intensity (A.U.)
D
n.s.
De novo WASP puncta/min
- EHT1864
+ EHT1864
E
n.s.
WASP puncta lifetime (s)
- EHT1864
+ EHT1864

Figure S3. Rac inhibition does not alter WASP dynamics nor its ability to trigger actin polymerisation on *puncta*. Related to Figure 4.

(A) Wild type cells expressing active Rac marker (PakB CRIB-mRFPmars2) were imaged prior to and upon addition of increasing concentration of the Rac inhibitor EHT1864. Before treatment (t=-10s), the active Rac marker is enriched on the plasma membrane, mostly at forming macropinosomes. Addition (t=0s) of low doses (0.3 μM, second line) of the inhibitor does not affect cell morphology nor the distribution of the active Rac marker. Addition (t=0s) of a higher dose (3 μM, third line) of the compound triggers a change in cell shape as well as a reduction of membrane-targeted active Rac marker. Further increase of the drug concentration (30 μM, bottom line) leads to a quicker and more dramatic effect. Scale bars=10 μm. As a control, cells were imaged prior to and upon addition of LoFlo, a low-fluorescence medium here utilised as buffer for the Rac inhibitor. As expected, adding LoFlo does not affect localisation of the active Rac marker, nor cell morphology (top line). (B) WASP's ability to generate F-actin-rich *puncta* upon chemical inhibition of Rac was investigated using Airyscan confocal imaging of *wasA*$^{-}$ cells expressing GFP-WASP and LifeAct-mRFPmars2. Before addition of the drug (t=-6s), WASP can be detected on individual spots (dashed square). Shortly after treatment (t=0s) with a high dose of the drug (50 μM), cells undergo a morphological change. As indicated by dashed squares (highlighted within insets) at given time-points, WASP is able to generate F-actin-rich *puncta* even after 11 minutes from the treatment. Scale bar=10 μm. (C) Left-hand side kymograph and fluorescence plot show a representative example of WASP generating an Arp2/3 complex-positive *punctum* in the presence of Rac inhibitor. Right-hand side kymograph and fluorescence plot report a representative example of WASP generating an F-actin-positive *punctum* in the presence of

Rac inhibitor. Both kymographs were obtained from a time-lapse as shown in B. (D) No significant difference can be measured between the number of WASP *puncta* generated per minute prior to and upon Rac inhibition. Untreated cells (-EHT1864, n=8 cells): 3.7 ± 1.0; treated cells (+EHT1864, n=10 cells): 2.7 ± 0.5; means ± SEM. Unpaired t-test, p=0.3130). (E) No significant difference can be measured between the lifetime of WASP *puncta* generated prior to and upon addition of the Rac inhibitor. Untreated cells (-EHT1864, n=57 *puncta*): 10.88s ± 0.59; treated cells (+EHT1864, n=213 *puncta*): 12.67s ± 0.49; means ± SEM. Mann-Whitney test, p=0.2023.

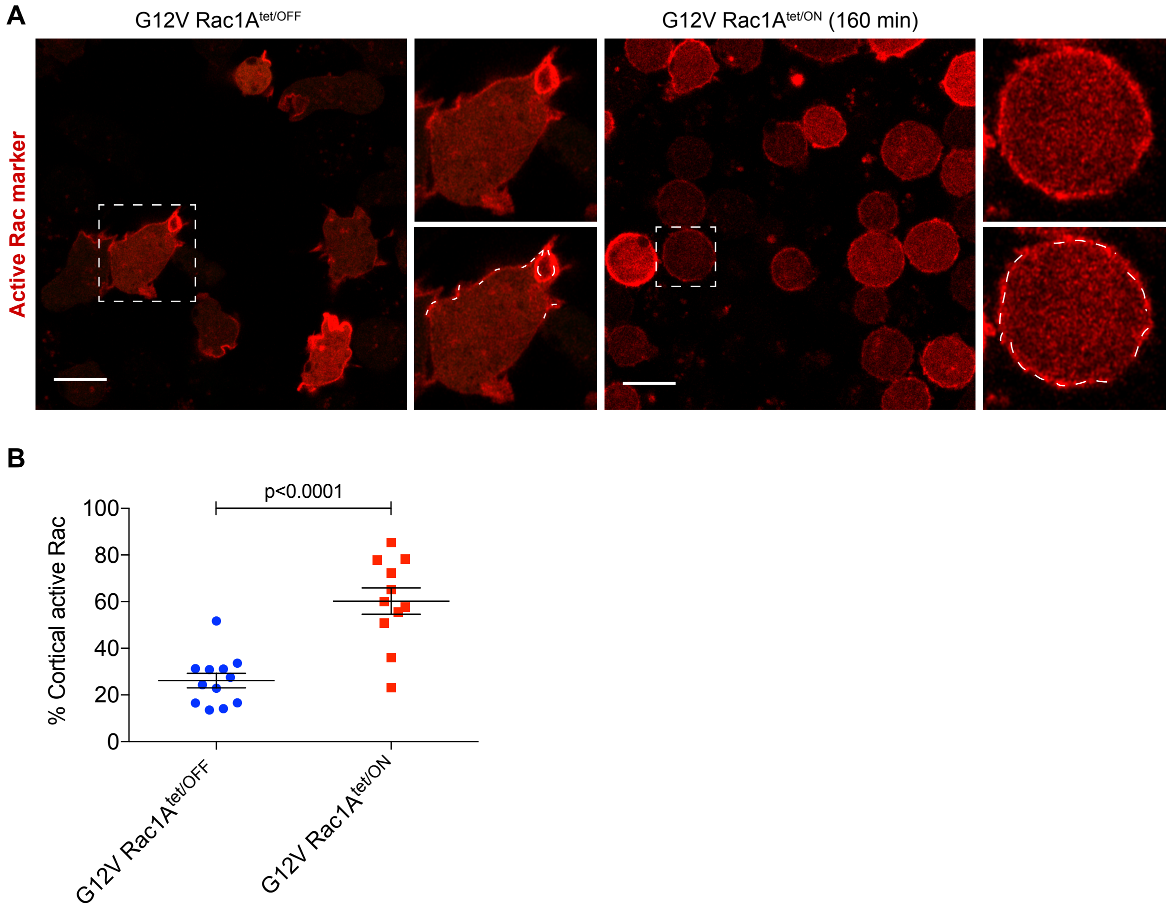

A
G12V Rac1A tet/OFF
G12V Rac1A tet/ON (160 min)
Active Rac marker
B
p<0.0001
% Cortical active Rac
100
80
60
40
20
0
G12V Rac1A tet/OFF
G12V Rac1A tet/ON

Figure S4. The proportion of plasma membrane labelled by the active Rac marker correlates with active Rac levels. Related to Figure 6.

(A) *wasA*$^-$ cells expressing GFP-WASP (rescue, green channel not shown), active Rac marker (pakB CRIB-mRFPmars2), and a tetracycline-inducible dominant active (G12V) Rac1A were imaged prior to and upon addition of tetracycline, and the percentage of plasma membrane labelled by the active Rac marker was measured. Prior to tetracycline treatment (left panels, G12V Rac1A$^{tet/OFF}$), the active Rac marker labels a small proportion of the plasma membrane. Upon addition of tetracycline (right panels, G12V Rac1A$^{tet/ON}$), cells accumulate the active Rac marker on a significantly larger proportion of their plasma membrane. (B) Quantification of the percentage (%) of plasma membrane labelled by the active Rac marker prior to and upon addition of tetracycline. - tetracycline (G12V Rac1A$^{tet/OFF}$, n=12 cells): 26.18% ± 3.12; +tetracycline (G12V Rac1A$^{tet/ON}$, n=11 cells): 60.25% ± 5.65; means ± SEM. Unpaired t-test, $p<0.0001$. Scale bars=10 μm.
