# Supplementary material for: WASP Restricts Active Rac to Maintain Cells’ Front-Rear Polarization
Source: Curr Biol. 2019 Dec 16;29(24):4169–4182.e4. doi: 10.1016/j.cub.2019.10.036 (PMC6926487; doi:10.1016/j.cub.2019.10.036)
Supplement: Document S2. Article plus Supplemental Information [file mmc5.pdf]

Article

Current Biology

WASP Restricts Active Rac to Maintain Cells' Front-Rear Polarization

Graphical Abstract

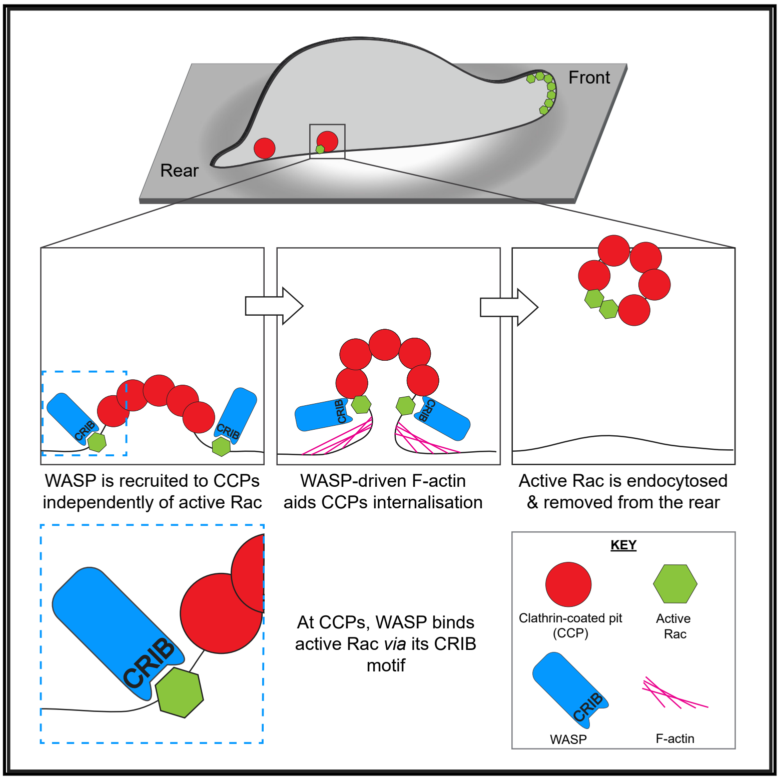

Highlights

- WASP exploits its CRIB motif to remove active Rac from the membrane via endocytosis
- WASP is recruited to clathrin-coated pits (CCPs) independently of small GTPases
- WASP triggers actin polymerization at CCPs independently of small GTPase activation
- WASP maintains homeostasis in *Dictyostelium* by controlling the level of active Rac

Authors

Clelia Amato, Peter A. Thomason, Andrew J. Davidson, Karthic Swaminathan, Shehab Ismail, Laura M. Machesky, Robert H. Insall

Correspondence

clelia.amato@ed.ac.uk

In Brief

Amato et al. have discovered a mechanism that contributes to front-rear polarization in migrating cells. During clathrin-mediated endocytosis, *Dictyostelium* WASP interacts with active Rac via its CRIB motif. This interaction leads to incorporation of active Rac within endocytic vesicles, which facilitates its removal from inappropriate locations.

Check for updates

Amato et al., 2019, Current Biology *29*, 4169–4182
December 16, 2019 © 2019 The Authors. Published by Elsevier Ltd.
https://doi.org/10.1016/j.cub.2019.10.036

WASP Restricts Active Rac to Maintain Cells' Front-Rear Polarization

Clelia Amato,[1,2,3,4,*] Peter A. Thomason,[1,2] Andrew J. Davidson,[1,2,3] Karthic Swaminathan,[1,2] Shehab Ismail,[1,2] Laura M. Machesky,[1,2] and Robert H. Insall[1,2]
[1]CRUK Beatson Institute, Switchback Road, Bearsden G61 1BD, UK
[2]Institute of Cancer Sciences, University of Glasgow, University Avenue, Glasgow G12 8QQ, UK
[3]Present address: Centre for Inflammation Research, the Queen's Medical Research Institute, University of Edinburgh, Edinburgh EH16 4TJ, UK
[4]Lead Contact
*Correspondence: clelia.amato@ed.ac.uk
https://doi.org/10.1016/j.cub.2019.10.036

SUMMARY

Efficient motility requires polarized cells, with pseudopods at the front and a retracting rear. Polarization is maintained by restricting the pseudopod catalyst, active Rac, to the front. Here, we show that the actin nucleation-promoting factor Wiskott-Aldrich syndrome protein (WASP) contributes to maintenance of front-rear polarity by controlling localization and cellular levels of active Rac. *Dictyostelium* cells lacking WASP inappropriately activate Rac at the rear, which affects their polarity and speed. WASP's Cdc42 and Rac interacting binding ("CRIB") motif has been thought to be essential for its activation. However, we show that the CRIB motif's biological role is unexpectedly complex. WASP CRIB mutants are no longer able to restrict Rac activity to the front, and cannot generate new pseudopods when SCAR/WAVE is absent. Overall levels of Rac activity also increase when WASP is unable to bind to Rac. However, WASP without a functional CRIB domain localizes normally at clathrin pits during endocytosis, and activates Arp2/3 complex. Similarly, chemical inhibition of Rac does not affect WASP localization or activation at sites of endocytosis. Thus, the interaction between small GTPases and WASP is more complex than previously thought—Rac regulates a subset of WASP functions, but WASP reciprocally restricts active Rac through its CRIB motif.

INTRODUCTION

Filamentous actin (F-actin) fulfils numerous functions in migrating cells. One crucial role is the generation of protrusions, such as pseudopods and lamellipods. A second is maintenance of intracellular trafficking, by driving endocytosis and vesicle sorting. These diverse functions depend upon the highly conserved Arp2/3 complex, which drives branching and growth of the actin network [1].

Cells rely on the Wiskott-Aldrich syndrome protein (WASP) family of nucleation-promoting factors (NPFs) to control the Arp2/3 complex spatially and temporally. The founding member of the family, WASP [2], is specific to hematopoietic lineages. Vertebrates possess a ubiquitous WASP paralog, N-WASP, which was originally described as a neural-specific gene [3] although expressed in nearly all cell types [4]. Other eukaryotes, including *Dictyostelium*, yeasts, and *Drosophila*, express a single WASP, which is the ortholog of vertebrates' ubiquitous isoform [5]. WASPs' principal role is to facilitate clathrin-mediated endocytosis (CME) [6–9].

Other members of the WASP family recruit and activate the Arp2/3 complex to other structures and for other purposes. For instance, the SCAR/WAVE complex drives formation of actin-rich protrusions [10–13] and is therefore an established regulator of cell migration and polarization.

Protrusions and clathrin-coated pits (CCPs) tend to occur in different regions of the cell. Protrusions can be generated almost anywhere on the membrane, but those that cause locomotion are usually initiated at the cell front, where SCAR/WAVE localizes. Whether CCPs are internalized in defined areas of the cell appears cell type dependent. In highly motile cells, including lymphocytes [14], leukocytes [15], and *Dictyostelium* [16], CME tends to occur at the rear.

WASP and SCAR/WAVE are normally spatially and functionally segregated. Nevertheless, they are plastic. In *Dictyostelium* and *Caenorhabditis*, WASP can compensate for loss of SCAR/WAVE [12, 17], generating actin protrusions at the cell front. Thus, WASP can respond to the upstream signals that drive pseudopod extension but does not normally do so. What determines segregation of WASP and SCAR/WAVE remains an important open question.

One prediction is that upstream regulators are crucial—distinct sub-cellular localization of activators may account for WASP's and SCAR/WAVE's distinct sub-cellular distribution and function. However, some of the upstream regulators appear to be shared between WASPs and SCAR/WAVE, for example, Nck [18, 19]. Small guanosine triphosphatases (GTPases) are also known crucial activators of both WASPs and SCAR/WAVE. However, while SCAR/WAVE has consistently been linked to Rac1 [20–22], there is no coherent model of how WASPs' behavior is regulated by small guanosine triphosphatases (GTPases).

This is an open access article under the CC BY license (http://creativecommons.org/licenses/by/4.0/).

WASPs from most organisms contain a "CRIB" (Cdc42/Rac interacting binding) motif, which binds selectively and specifically to the active form of Rac and Cdc42 [23] in many different proteins [23, 24]. Early *in vitro* measurements concluded that Cdc42 had a major role in activating N-WASP [4, 25]. However, more recent and precise biochemical analysis suggests that Cdc42 activates hematopoietic WASP, while Rac1 also interacts with N-WASP [26]. For *Dictyostelium* WASP, most attention has focused on the unusual RacC [27], although WASP also efficiently interacts with members of the Rac1 subfamily [27], which are more abundant (http://dictyexpress.biolab.si) and more closely related to mammalian Cdc42 and Racs. The *Dictyostelium* genome contains genes for many Rac relatives but no Cdc42 [28].

Understanding spatial and functional segregation of WASPs and SCAR/WAVE requires an improved comprehension of interactions with small GTPases. In fact, a model whereby Rac mediates the activation of both NPFs fits poorly with their distinct sub-cellular localization and functionality.

Recent work offers a fresh perspective on how cells maintain spatial and functional separation of WASP and SCAR/WAVE [6]. Loss of WASP in *Dictyostelium* causes aberrant accumulation of SCAR/WAVE at the rear, leading to defective retraction [6] and compromised cell polarity. Here, we dissect the role for WASP in maintenance of front-rear polarization.

We demonstrate that WASP exploits its CRIB-mediated interaction with active Rac to limit where the active GTPase is found. Furthermore, this work clarifies the importance of GTPases for WASP's function: a direct interaction with active GTPases is not needed for WASP to trigger actin polymerization during CME, but is required for WASP to generate pseudopods in SCAR/WAVE's absence. More provocatively, our study suggests a reversed role for the interaction between WASP and GTPases: the presence of a CRIB motif does not only mean that WASP activity requires GTPase regulation, but that WASP modulates the distribution of GTPases after they are activated.

RESULTS

Loss of WASP Causes Accumulation of SCAR/WAVE and Active Rac at the Rear

Previous work shows that knockout mutants in the *Dictyostelium* gene encoding WASP, *wasA*, migrate slower than their wild-type counterparts, due to a defect in rear retraction [6]. Detailed analysis revealed that, although wild-type cells confine SCAR/WAVE at the extending protrusions (Figure 1A), *wasA*$^{-}$ cells also accumulate it within an enlarged rear (Figure 1B). Mutants fail to confine SCAR/WAVE to a single region of their plasma membrane, resulting in a nearly bipolar appearance. Here, we examined the drivers of *wasA*$^{-}$ cells' inability to exclude SCAR/WAVE from their rear.

One intriguing possibility was that WASP may be responsible for the spatial restriction of SCAR/WAVE's activators, preventing actin polymerization in unwanted areas of the cell. Because active Rac is one of the major SCAR/WAVE regulators [20, 21, 29], we asked whether localization or dynamics of the GTP-bound GTPase was affected in *wasA*$^{-}$ cells. We imaged living cells expressing fluorescently tagged CRIB motif of PakB, which binds to many *Dictyostelium* Racs, including Rac1A-C and RacC, with high-affinity *in vitro* [30] and is thus an effective reporter for active Rac [12]. Similar constructs have been used to monitor endogenous active Rac in mammalian cells [31–33]. As expected, wild-type cells accumulate active Rac at the leading edge (Figures 1C and S1A), where it co-localizes with F-actin. *wasA*$^{-}$ cells also accumulate active Rac at the enlarged rear (Figures 1D and S1B; Video S1), leading to the formation of F-actin-rich structures at both ends. Quantification revealed that, although wild-type cells occasionally show active Rac at the rear, mostly when pseudopods are swept from the front of the cell as it moves, *wasA*$^{-}$ cells more frequently accumulate active Rac at their backs (Figure 1E). Because the localization of both active Rac and SCAR/WAVE is aberrant in *wasA*$^{-}$ cells and Rac directly regulates SCAR/WAVE, the simplest conclusion would be that WASP limits the amount of membrane with active Rac.

Generation of WASP CRIB Mutants

We tested possible molecular mechanisms underlying WASP-mediated confinement of active Rac and SCAR/WAVE. We focused on WASP's CRIB motif, which enables WASP to bind active GTPases, by introducing mutations (Figure 2A). The first mutant, WASP$^{\Delta CRIB}$, harbors a deletion of 14 residues (173–186) of the CRIB's core [23]. A related mutant has been described in *Drosophila* [34]. To ensure this substantial deletion did not affect function, we designed a second mutant (WASP^{**CRIB}), containing only two conservative amino acidic changes (I173A; F179A), chosen for their position in the WASP/Rac interface (Figure 2B), inferred from the structure of the complex between Cdc42 and WASP's minimal p21 binding domain, which includes the CRIB motif [35]. Changing them to alternative hydrophobic amino acids should maximally diminish the binding energy, with minimal change to the CRIB motif's structure. Importantly, both changes affect the N terminus of the CRIB motif, which is not primarily involved in maintenance of the autoinhibited conformation [36]. We therefore do not expect these mutations to steer WASP to an inappropriately active conformation.

We tested the two WASP CRIB mutants' interaction with active Rac using pull-down assays. We examined Rac1A, highly expressed and closely related to canonical Rac1 of other species, and RacC, which has been associated with *Dictyostelium* WASP [27]. Bacterially purified glutathione S-transferase (GST)-Racs were activated by loading with GTPγs, a non-hydrolyzable analog of GTP. Wild-type WASP binds to active Racs, but neither CRIB mutant is able to bind either GTPase (Figures 2C, 2D, and S2).

Rac Binding Is Not Required to Recruit WASP to CCPs

The most widely accepted model of WASP regulation asserts that Rac or Cdc42 have an essential role in controlling WASP's activity [4, 25–27], implying WASP mutants that cannot interact with active Rac should lose function. To test which physiological functions of WASP depend on Rac regulation and a functional CRIB domain, we imaged *wasA*$^{-}$ cells co-expressing fluorescently tagged WASP mutants and clathrin.

As shown in Figures 3A and 3B, wild-type WASP generates *puncta* that overlap with CCPs. At any time, the number of clathrin *puncta* that are WASP positive is low; this is because

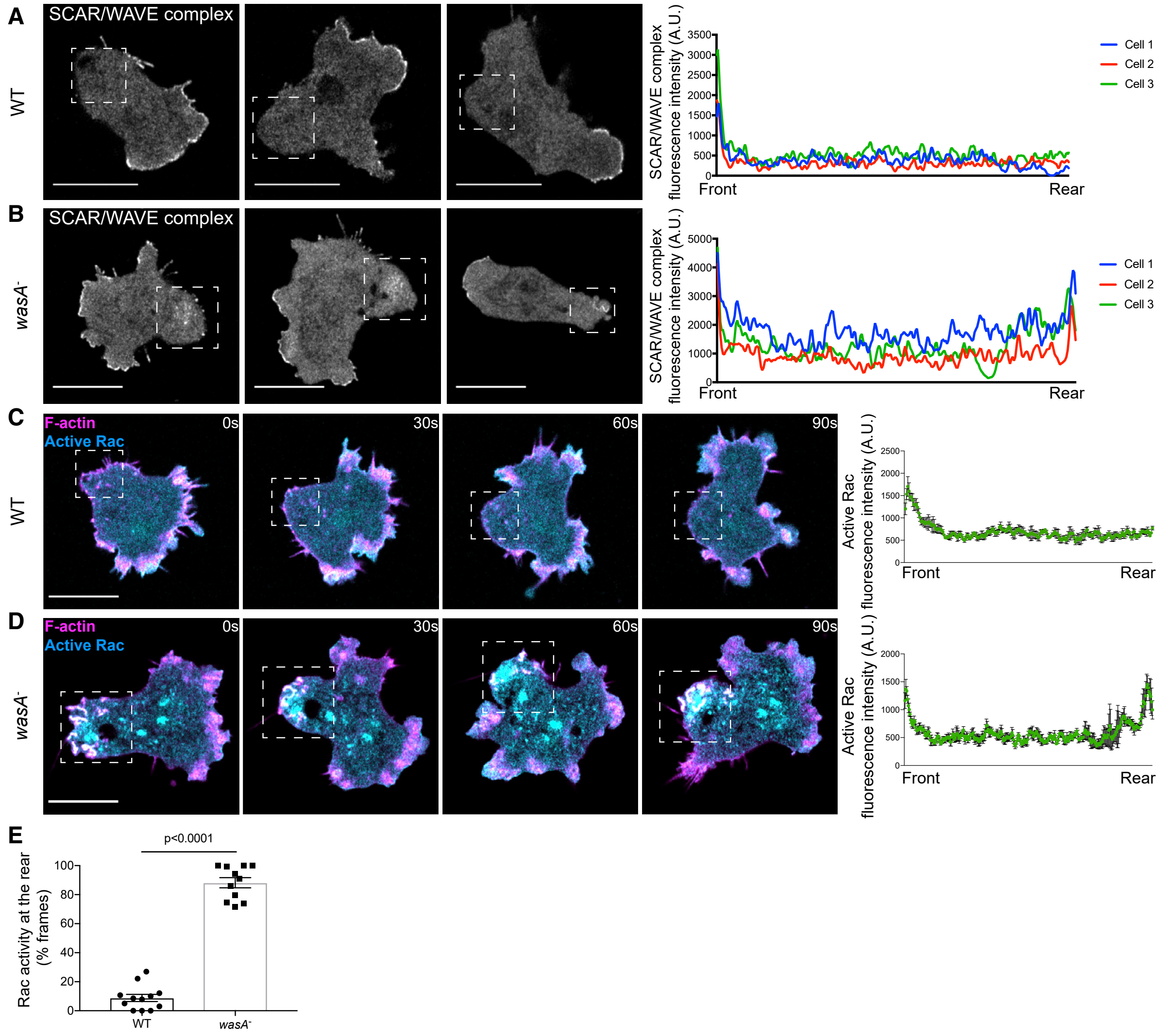

Figure 1. *wasA*⁻ Cells Accumulate SCAR/WAVE and Active Rac at the Rear

(A and B) SCAR/WAVE (HSPC300-GFP) localizes at the front but not at the rear (squares) of migrating wild-type (WT) cells (A) but is present at both ends in *wasA*⁻ cells (B). Fluorescence plots on the right-hand side report the SCAR/WAVE's intensity along a line connecting front to rear of representative cells at a given time point. Scale bars represent 10 μm.

(C and D) Active Rac (PakB CRIB-GFP) is confined to the front and excluded from the rear (squares) of migrating WT cells (C) but is present at the enlarged rear (squares) of *wasA*⁻ cells (D); see also Figure S1. Related to Video S1. Fluorescence plots show active Rac enrichment along a line connecting front to rear of individual cells at four time points. F-actin (LifeAct-mRFP) is detected at the front and on transient spots at the rear (squares) of WT cells (C) and at the front but also on persistent structures at the rear (squares) of *wasA*⁻ cells (D). Scale bars represent 10 μm.

(E) Frequency (% frames; yes/no scoring) of active Rac enrichment at the rear of migrating WT and *wasA*⁻ cells. WT (n = 12 cells): 8.9 ± 2.5; *wasA*⁻ (n = 11 cells): 88.2 ± 3.5; means ± SEM. Mann-Whitney test; $p < 0.0001$.

WASP appears in a brief burst while CCPs persist on the plasma membrane for a longer period of time. Figure 3C shows a representative example of clathrin/WASP dynamics visualized using TIRF (total internal reflection fluorescence) microscopy of cells gently compressed under agarose, which diminishes front-rear polarity. WASP is recruited on a pre-existing CCP, where it sits for a few seconds before disappearing along with clathrin. Clearance of WASP and clathrin from the TIRF field is considered as a *bona fide* sign of CCP internalization [37]. To our surprise, both WASP CRIB mutants generate *puncta* that coincide with CCPs (Figures 3D, 3E, 3G, and 3H). Live TIRF imaging confirmed that $WASP^{\Delta CRIB}$ and $WASP^{**CRIB}$ are recruited to pre-existing clathrin *puncta* (Figures 3F and 3I).

This clearly demonstrates that a direct interaction with active Rac is not required for WASP to be recruited to CCPs, suggesting that factors other than GTPases control WASP's sub-cellular localization in living cells during endocytosis.

WASP Does Not Require Rac to Recruit Arp2/3 Complex and Actin

We noticed that the WASP CRIB mutants co-localize with clathrin for longer than wild-type WASP. This could be caused by

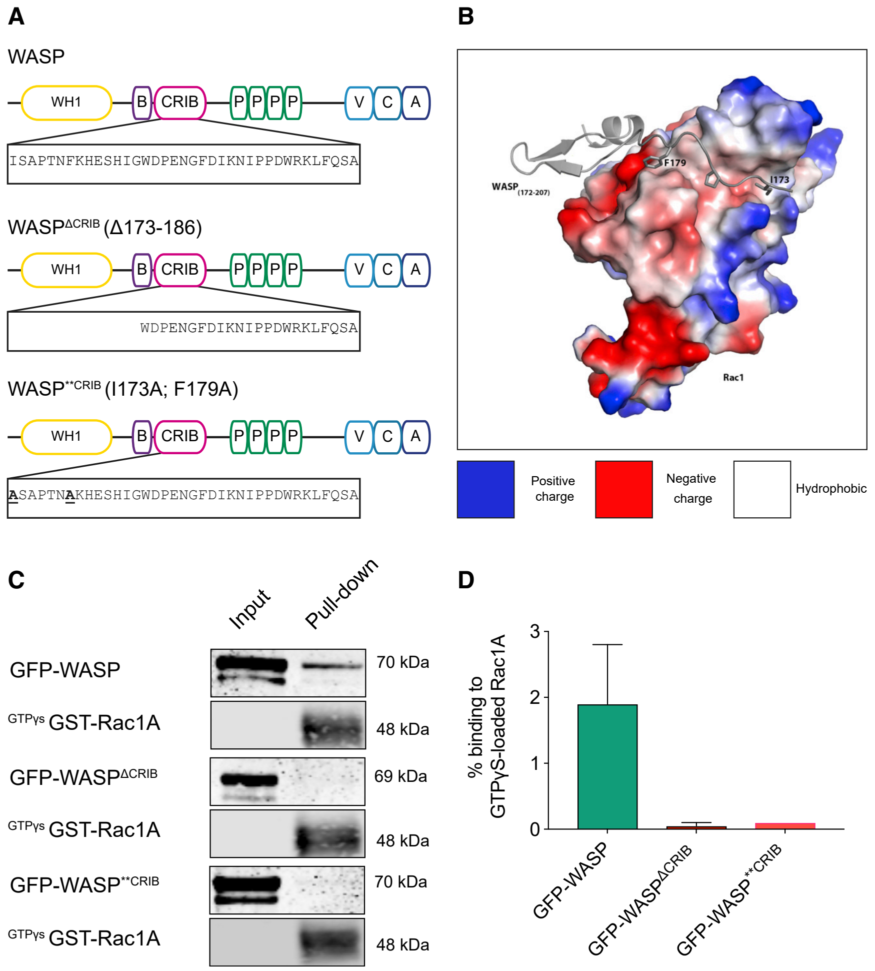

Figure 2. Mutations in the WASP CRIB Motif Abrogate Binding to Active Rac1

(A) WASP domain composition and mutations introduced within the CRIB motif. From top to bottom: WASP; WASP$^{\Delta CRIB}$; and WASP^{**CRIB} are shown.

(B) 3D representation of WASP/Rac1 interface. WASP (gray) residues I173 and F179 establish contacts with a hydrophobic (white) region of Rac1.

(C) GFP-WASP (first panel) interacts with active (GTPγs-bound) Rac1A, GFP-WASP$^{\Delta CRIB}$ and GFP-ASP^{**CRIB} (third and fifth panels) do not (IB = anti-GFP). Anti-GST immunoblot was performed (second, fourth, and sixth panels) to verify the expression of GST-Rac1A. Related to Figure S2.

(D) Immunoblot quantification shows no binding of GFP-WASP$^{\Delta CRIB}$ and GFP-WASP^{**CRIB} to active Rac1 (GFP-WASP: 1.9% ± 0.9%; GFP-WASP$^{\Delta CRIB}$: 0.05% ± 0.05%; GFP-WASP^{**CRIB}: 0.1; mean ± SEM; n = 3).

their inability to trigger actin polymerization on CCPs, as F-actin is thought to provide the force required for CME [38, 39] and is mandatory in yeast and *Dictyostelium* [6, 40], but not all mammalian cells [41, 42]. Given our demonstration that loss of WASP causes increased CCP lifetime due to the lack of actin (Figure 2E in [6]), we tested whether WASP CRIB mutants are able to trigger actin polymerization once recruited to CCPs. In essentially 100% of cases, WASP spots (which tend to be distributed over the rear half of polarized cells) are Arp2/3 complex positive, presumably reflecting the fact that WASP is activated as it is targeted to CCPs, so the Arp2/3 complex is recruited synchronously (Figures 4A and 4B). Unexpectedly, 100% of *puncta* generated by mutated WASPs are also Arp2/3 complex positive (Figures 4C–4F). Spots generated by WASP$^{\Delta CRIB}$ and WASP^{**CRIB} were more likely to be seen within the enlarged rear of the cell, with increased lifetimes (Figure 4G). Consistent with the Arp2/3 complex data, F-actin was observed on all WASP *puncta* irrespective of the presence of a functional CRIB domain (Figure 4H; Video S2). WASP's ability to drive actin polymerization at CCPs is therefore independent of small GTPase binding.

For further experimental support, we tested WASP's activity upon chemical inhibition of Rac. Because no Rac inhibitor had been verified for *Dictyostelium*, we tested candidates using wild-type cells expressing an active Rac marker. NSC23766 [43] failed to show any effect (not shown). However, EHT1864, which blocks Rac's loading with GTP in mammalian cells [44, 45], was very effective in *Dictyostelium* (Figure S3A). A minimal dose of 3 μM causes rapid loss of membrane localization of the active Rac marker, leading to progressive cell rounding.

In agreement with our data highlighting the ability of CRIB-mutated WASP to be recruited to CCPs, we found that WASP is able to generate Arp2/3-complex- and F-actin-positive *puncta* after Rac inhibition (Figures 4I, S3B, and S3C). Quantitative analysis (Figures S3D and S3E) shows that EHT1864 does not alter the rate at which WASP *puncta* are generated or their lifetime. This result reinforces our conclusion that WASP does not need active GTPases to localize correctly during CME or to recruit Arp2/3 complex and generate F-actin.

WASP Interaction with Active Rac Is Essential for Front-Rear Polarity

The generation of WASP CRIB mutants allowed us to test whether WASP requires a direct interaction with active Rac to maintain front-rear polarization.

wasA^{-} cells accumulate active Rac at the leading edge, but also within the enlarged rear (Figures 5A and 1D). *wasA*$^{-}$ cells expressing GFP-tagged WASP (rescue) behave normally, accumulating active Rac exclusively at the front (Figure 5B). CRIB-mutated WASPs do not suppress the accumulation of active Rac at the rear—both mutants generate discrete *puncta* that accumulate within the enlarged rear, together with persistent active Rac-marker-positive structures like those seen in unrescued *wasA*$^{-}$ cells (Figures 5C and 5D; Video S3). At a given time, a subset of *puncta* in WASP CRIB mutants is also enriched

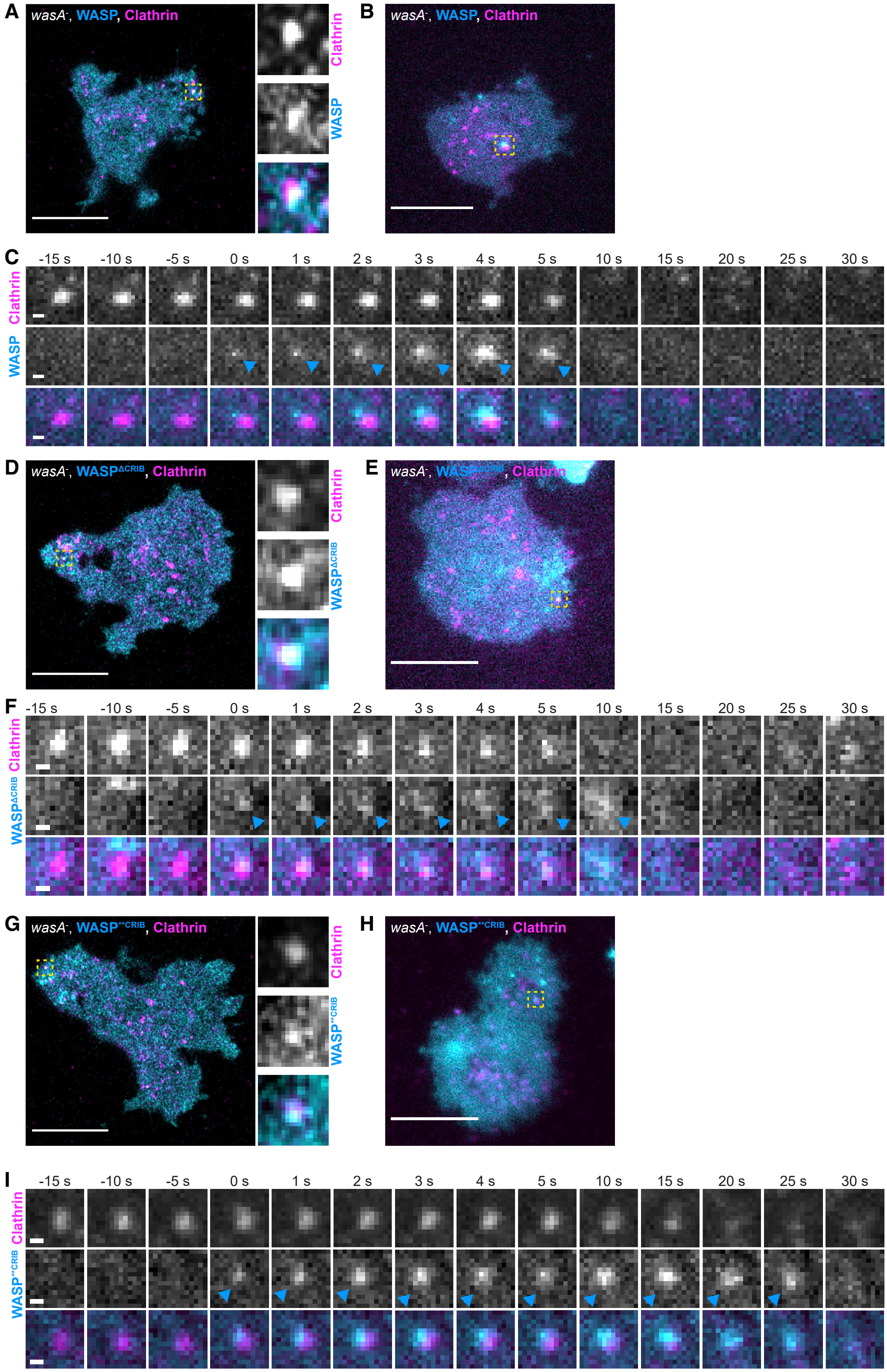

Figure 3. WASP Does Not Require a Direct Interaction with Active Rac to Localize to CCPs

(A) Live imaging of migrating *wasA*⁻ cells co-expressing WASP (GFP-WASP, rescue) and clathrin light chain (clc)-mRFPmars. Example of clathrin/WASP co-localization is indicated (square) and highlighted (insets). Scale bar represents 10 μm.

(legend continued on next page)

in active Rac. WASP-CRIB-mutants and active Rac appear to have distinct dynamics: active Rac appears to remain at the plasma membrane for longer than CRIB-mutated WASPs. Therefore, although some active Rac-positive structures are not enriched in CRIB-mutated WASPs, we cannot exclude that they were enriched in CRIB-mutated WASP at some stage. Representative plots showing active Rac marker localization from front to rear are shown in Figure 5E. Quantification (Figure 5F) reveals that cells expressing CRIB-mutated WASPs accumulate active Rac at the rear to the same degree as *wasA*$^-$ cells, while wild-type WASP rescues the spatial confinement of active Rac at the leading edge. In line with previous reports from *wasA*$^-$ cells [6], cells expressing either WASP CRIB mutant are significantly slower than those expressing wild-type WASP (Figure 5G).

Our results show that WASP requires an intact CRIB motif to exclude active Rac from the cell rear during migration.

WASP Contributes to Homeostasis of Active Rac Levels

We examined whether WASP works locally, to remove active Rac from inappropriate locations, or has a more global role in Rac regulation.

Taking into account that active Rac is largely membrane bound [46], and that the CRIB-based marker interacts selectively with GTP-bound Rac [30] but is cytosolic when unbound, then the ratio of active Rac marker at the membrane and in the cytosol offers a measure of the total levels of active Rac in the cell [12]. We therefore measured the relative quantities of active Rac marker at the membrane and within the cytosol in cells expressing CRIB-mutated WASPs. As shown in Figure 6A (quantified in Figure 6B), the membrane:cytosol ratio of the active Rac marker is consistently and significantly higher in cells expressing a CRIB-mutated WASP in comparison with cells expressing wild-type WASP. We confirmed this result by pulling down the active Rac from cell lysates using GST-CRIB beads (Figure 6C). Again, we observed a higher level of active Rac in cell expressing WASP CRIB mutants.

We also examined the extent of active Rac patches. Cells expressing CRIB-mutated WASPs accumulate the active Rac marker in a significantly larger portion of their plasma membrane than cells expressing wild-type WASP (Figures 6D and 6E). This increase in active Rac localization is accompanied by a reduction in cell polarity (Figure 6F). Cells expressing wild-type WASP are elongated even when migrating randomly, while those expressing either WASP CRIB mutant are more rounded, with a length:width ratio of nearly one. No significant difference between the average perimeter of cells expressing wild-type or CRIB-mutated WASPs was seen (Figure 6G), so the more widespread localization of the active Rac marker in cells expressing WASP CRIB mutants is not due to the availability of more plasma membrane. We confirmed the correlation between active Rac levels and the proportion of plasma membrane labeled by the active Rac marker using tetracycline-inducible dominant-active (G12V) Rac1A. Induction with tetracycline causes cells to accumulate the active Rac marker on a higher proportion of the plasma membrane, often close to 100% (Figures S4A and S4B).

Altogether, our data show that cells whose WASP lacks a functional CRIB motif, and thus cannot bind Rac, accumulate more active Rac on a higher portion of the plasma membrane than cells with an intact WASP.

WASP Requires Active Rac Interaction to Replace SCAR/WAVE at Pseudopods

Internalization of CCPs is by far the clearest and evolutionarily conserved role of WASP; however, WASP can contextually fulfil other biological roles. For instance, WASP can drive protrusion extension in cells that have lost SCAR/WAVE [12]. We therefore asked whether WASP can drive pseudopods extension independently of Rac1, using a cell line in which *wasA* is knocked out and SCAR expressed under a tetracycline-inducible promoter (*wasA*$^-$ *scrA*^{tet}). We expressed CRIB-mutated GFP-WASP in *wasA*$^-$ *scrA*^{tet} cells and examined their ability to migrate toward chemoattractant in the presence of tetracycline (when SCAR/WAVE is expressed) or without (when the only pseudopod inducer was the exogenously expressed WASP).

As shown in Figure 7A, cells expressing SCAR/WAVE and wild-type WASP (*wasA*$^-$ *scrA*$^{tet/ON}$/GFP-WASP) migrate efficiently in a chemotactic gradient. In these cells, WASP localizes to F-actin-rich *puncta*. Once the expression of SCAR/WAVE is turned off (Figure 7B), cells expressing wild-type WASP (*wasA*$^-$ *scrA*$^{tet/OFF}$/GFP-WASP) are still able to move effectively. In these cells, WASP is additionally localized to the leading edge, where it drives formation of pseudopods, as previously described [12]. Cells expressing WASP$^{\Delta CRIB}$ or WASP^{**CRIB}

(B) TIRF microscopy of *wasA*$^-$ cells co-expressing GFP-WASP (rescue) and clc-mRFPmars gently compressed under agarose. Example of clathrin/WASP co-localization is indicated (square). Scale bar represents 10 μm.

(C) TIRF imaging shows GFP-WASP appearing (t = 0 s, cyan arrowhead) on a pre-existing CCP. Clathrin and WASP disappear synchronously (t = 10 s). Scale bars represent 0.5 μm.

(D) Live imaging of migrating *wasA*$^-$ cells co-expressing GFP-WASP$^{\Delta CRIB}$ and clc-mRFPmars. Example of clathrin/WASP$^{\Delta CRIB}$ co-localization is indicated (square) and highlighted (insets). Scale bar represents 10 μm.

(E) TIRF microscopy of *wasA*$^-$ cells co-expressing GFP-WASP$^{\Delta CRIB}$ and clc-mRFPmars gently compressed under agarose. Example of clathrin/WASP$^{\Delta CRIB}$ co-localization is indicated (square). Scale bar represents 10 μm.

(F) TIRF imaging shows GFP-WASP$^{\Delta CRIB}$ appearing on a pre-existing CCP (t = 0 s, cyan arrowhead). Clathrin and GFP-WASP$^{\Delta CRIB}$ disappear synchronously (t = 15 s). Scale bars represent 0.5 μm.

(G) Live imaging of migrating *wasA*$^-$ cells co-expressing GFP-WASP^{**CRIB} and clc-mRFPmars. Example of clathrin/WASP^{**CRIB} co-localization is indicated (square) and highlighted (insets). Scale bar represents 10 μm.

(H) TIRF microscopy of *wasA*$^-$ cells co-expressing GFP-WASP^{**CRIB} and clc-mRFPmars gently compressed under agarose. Example of clathrin/WASP^{**CRIB} co-localization is indicated (square). Scale bar represents 10 μm.

(I) TIRF imaging shows GFP-WASP^{**CRIB} appearing on a pre-existing CCP (t = 0 s, cyan arrowhead). Clathrin and GFP-WASP^{**CRIB} disappear from the TIRF field synchronously (t = 25 s). Scale bars represent 0.5 μm.

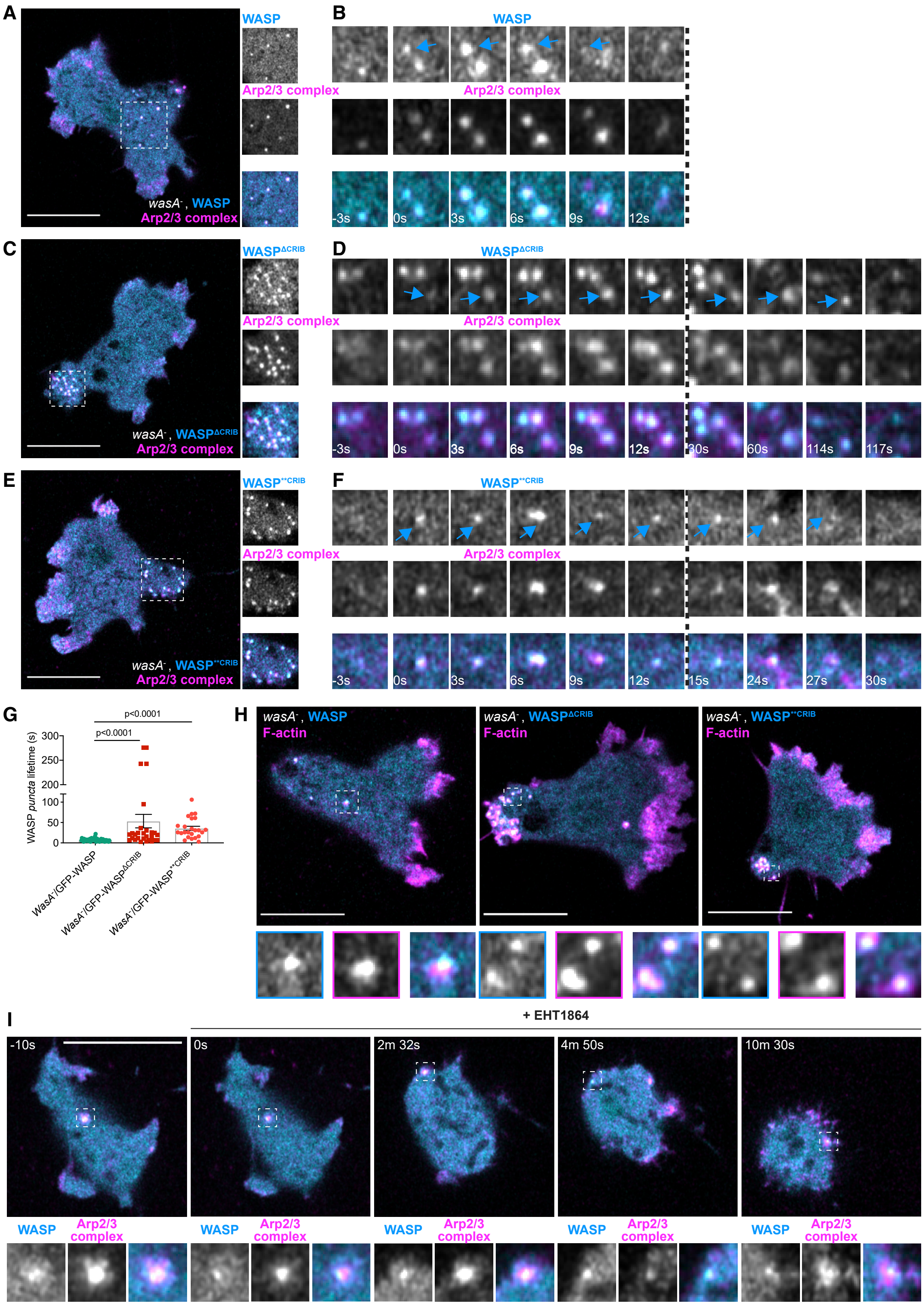

(legend on next page)

migrate fairly efficiently toward a chemoattractant as long as the expression of SCAR/WAVE is maintained (Figures 7C and 7E; *wasA*$^{-}$ *scrA*$^{tet/ON}$/GFP-WASP$^{\Delta CRIB}$ and *wasA*$^{-}$ *scrA*$^{tet/ON}$/GFP-WASP^{**CRIB}, respectively). In each case, the mutated WASPs localize to *puncta* clustered within the enlarged rear. When expression of SCAR/WAVE is turned off, cells expressing either WASP CRIB mutant lose the ability to move, highly resembling cells lacking SCAR and WASP [6] (Figures 7D and 7F; *wasA*$^{-}$ *scrA*$^{tet/OFF}$/GFP-WASP$^{\Delta CRIB}$ and *wasA*$^{-}$ *scrA*$^{tet/OFF}$/GFP-WASP^{**CRIB}, respectively). Under these circumstances, pseudopods are no longer generated but replaced by inefficient spiky protrusions, which cannot translocate the cell.

Thus, a functional CRIB motif—and therefore a direct activation by Rac—is essential for WASP to stimulate pseudopod formation in the absence of SCAR/WAVE.

DISCUSSION

WASP's Interaction with Small GTPases: Implications for WASP Regulation

Considering how important actin nucleation promoting factors are, for processes as diverse as cell movement [10–12], macropinocytosis [47], vesicular sorting [48, 49], mitosis [50], meiosis [51], and DNA repair [52], their control is exceptionally poorly understood. The SCAR/WAVE complex contains five proteins [21] and harbors multiple potential phosphorylation and protein-protein interaction sites [53], but the relevance of most is largely unknown. WASPs, the family that includes mammalian WASP and N-WASP, the singular *Dictyostelium* and *Drosophila* WASP, were thought to be an exception. Largely based on biochemical data, the interaction with active GTPases (Cdc42 or Rac) has long been considered essential for WASP activation. Other factors, such as PIP_2 and adaptor proteins, may contribute to the NPFs activation [3, 4, 25, 26, 54–56]. But *in vitro* settings do not recapitulate the complexity of a living cell, and early data implying a key role for small GTPases in WASP activation have not been corroborated in living cells. Studies in *Drosophila* suggest that CRIB-mutated WASP can still fulfil its developmental roles [34], and a CRIB-deleted N-WASP still triggers actin comet tails formation in PIP5K-overexpressing cultured fibroblasts [57]. Evolutionary arguments point in the same direction: yeasts' WASPs have lost their Rac-binding CRIB motif during evolution but facilitate CME by a remarkably similar mechanism to higher eukaryotes' WASPs [8].

In other words, although a number of reviews describe GTPase binding as an essential step toward WASP localization and activation [58, 59], our understanding of this interaction's importance to living cells is still poor. Our work adds to this complex jigsaw. By introducing a deletion within the WASP CRIB motif or—importantly—a finer mutation that abrogates interaction with Rac using only two conservative substitutions, we show that WASP does not rely on GTPases to localize to CCPs nor to induce Arp2/3-complex-mediated actin polymerization once there.

WASP's Role in Front-Rear Polarity

If WASP does not need GTPases to localize to CCPs or to trigger actin polymerization on *puncta*, why does it interact with them? CRIB motifs are the best-understood GTPase interactors, with high specificity for the active forms of Rac and Cdc42 [23]. *Dictyostelium* lacks a Cdc42 [28], which first appears just before fungal and metazoan evolutionary lineages divide, but its WASP has a well-conserved CRIB motif that gives strong binding specificity to active Rac1A and C [27]. What are its physiological roles?

We provide two related answers. First, WASP needs a direct interaction with active Rac to compensate for loss of SCAR/WAVE. WASPs are not normally involved in pseudopod formation [60] but take over when SCAR/WAVE is absent [17]. Because active Rac is the key specifier of the leading edge, it is unsurprising that WASP requires Rac binding to do so.

The second role for Rac binding to WASP is more subtle. WASP exploits its CRIB motif to prevent aberrant accumulation of the GTPase at the cell rear. When there is no WASP, cells lose polarity in an idiosyncratic way—they generate pseudopods at the front but also carry a substantial, complex assembly at the rear that slows and depolarizes them. This aggregate contains active Rac and therefore continuously recruits SCAR/WAVE and Arp2/3 complex, inducing actin polymerization. This raises two main

Figure 4. WASP Does Not Require Active Rac to Recruit the Arp2/3 Complex and Trigger Actin Polymerization on *Puncta*

(A) Live imaging of migrating *wasA*$^{-}$ cells co-expressing GFP-WASP (rescue) and mRFPmars2-ArpC4. WASP *puncta* are evenly distributed at the rear and are all Arp2/3 complex positive. The area indicated by the square is highlighted within insets. Scale bar represents 10 μm.

(B) GFP-WASP appears on a *punctum* (t = 0 s, cyan arrow) alongside mRFPmars2-ArpC4 and then synchronously disappears (t = 9 s).

(C) Live imaging of migrating *wasA*$^{-}$ cells co-expressing GFP-WASP$^{\Delta CRIB}$ and mRFPmars2-ArpC4. All WASP$^{\Delta CRIB}$ *puncta* cluster within the enlarged rear (square). As highlighted (insets), all GFP-WASP$^{\Delta CRIB}$ spots are Arp2/3 complex positive. Scale bar represents 10 μm.

(D) GFP-WASP$^{\Delta CRIB}$ appears as a *punctum* (t = 0 s, cyan arrow) along with mRFPmars2-ArpC4. WASP$^{\Delta CRIB}$ remains within spots (alongside the Arp2/3 complex) and then synchronously disappears (t = 117 s).

(E) Live imaging of migrating *wasA*$^{-}$ cells co-expressing GFP-WASP^{**CRIB} and mRFPmars2-ArpC4. All WASP^{**CRIB} *puncta* cluster within the enlarged rear (square). As shown in insets, all WASP$^{\Delta CRIB}$ spots are Arp2/3 complex positive. Scale bar represents 10 μm.

(F) GFP-WASP^{**CRIB} appears as a *punctum* (t = 0 s, cyan arrow) alongside mRFPmars2-ArpC4. They disappear synchronously (t = 27 s).

(G) Lifetime of wild-type and WASP CRIB mutants' *puncta*. *wasA*$^{-}$/GFP-WASP (n = 52 *puncta*): 7.2 ± 0.5 s; *wasA*$^{-}$/GFP-WASP$^{\Delta CRIB}$ (n = 28 *puncta*): 53.4 ± 16.6 s; *wasA*$^{-}$/GFP-WASP^{**CRIB} (n = 24 *puncta*): 35.8 ± 5.1 s; means ± SEM. Kruskal-Wallis test, *wasA*$^{-}$/GFP-WASP versus *wasA*$^{-}$/GFP-WASP$^{\Delta CRIB}$ = $p < 0.0001$; *wasA*$^{-}$/GFP-WASP versus *wasA*$^{-}$/GFP-WASP^{**CRIB} = $p < 0.0001$).

(H) Live imaging of migrating *wasA*$^{-}$ cells expressing LifeAct-mRFPmars2 and GFP-WASP (rescue, left), GFP-WASP$^{\Delta CRIB}$ (center), and GFP-WASP^{**CRIB} (right). All *puncta* generated by wild-type WASP or WASP CRIB are F-actin positive. Examples indicated by squares are highlighted within insets. Scale bars represent 10 μm. Related to Video S2.

(I) Live imaging of *wasA*$^{-}$ cells expressing GFP-WASP and mRFPmars2-ArpC4 treated with Rac inhibitor (EHT1864). Before EHT1864 addition (t = −10 s), cells are polarized and WASP generates Arp2/3-complex-positive *puncta* (square). Shortly after treatment (t = 0 s), cells become rounder. As highlighted by squares, WASP generates Arp2/3-complex-positive *puncta* even after 10 min from treatment. Scale bar represents 10 μm.

See also Figure S3.

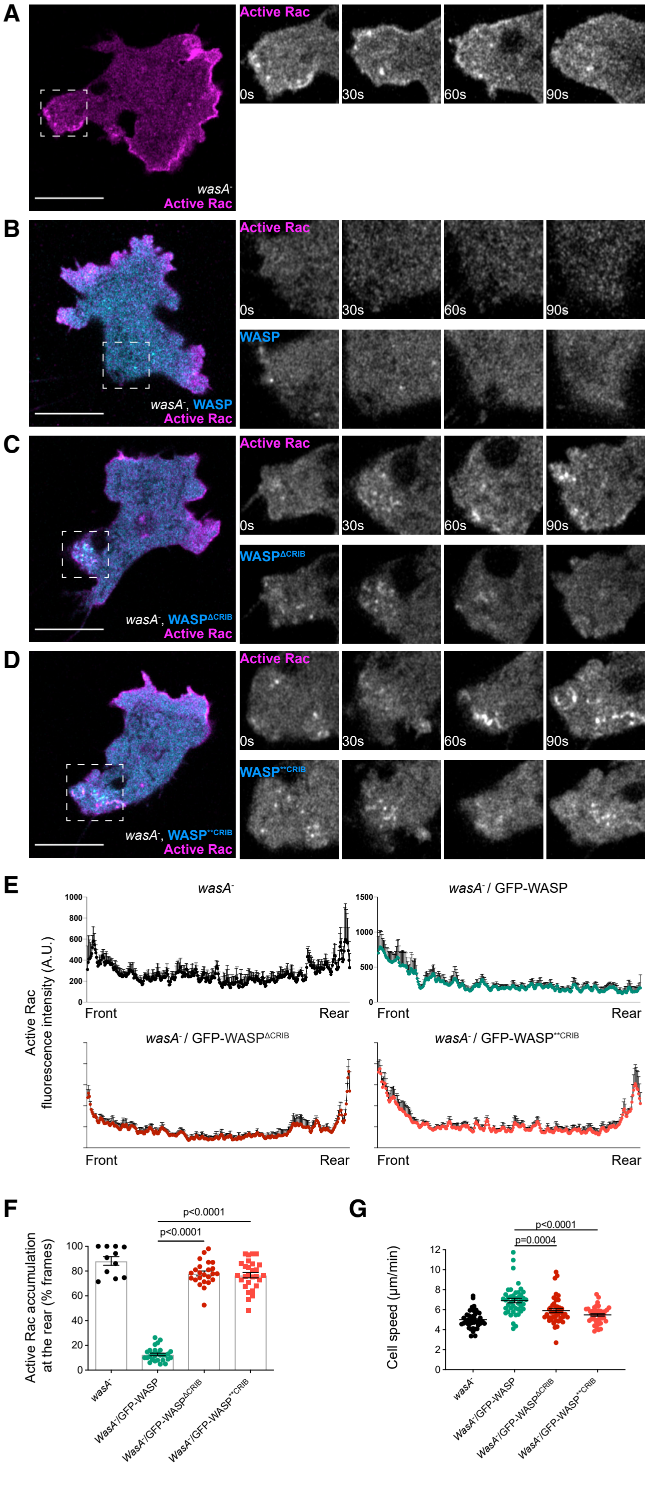

Figure 5. WASP Requires a Functional CRIB Motif to Confine Active Rac at the Leading Edge during Migration

Live imaging of migrating *wasA*⁻ cells expressing active Rac marker (PakB CRIB-mRFPmars2). Insets highlight the cells' rear (squares) at different time points.

(A) Active Rac accumulates at the rear of *wasA*⁻ cells at all time points. Scale bar represents 10 μm.

(B) In cells expressing GFP-WASP, no sign of active Rac enrichment at the rear can be detected. Scale bar represents 10 μm.

(C and D) In cells expressing GFP-WASP$^{\Delta CRIB}$ (C) or GFP-WASP^{**CRIB} (D), active Rac can be detected within the enlarged rear. *Puncta* generated by WASP CRIB mutants cluster at the enlarged rear. Scale bars represent 10 μm. Related to Video S3.

(E) Fluorescence plots reporting the active Rac marker's intensity at four time points along a line connecting front to rear of *wasA*⁻ cells (top left), and *wasA*⁻ cells expressing either WASP (*wasA*⁻/GFP-WASP, top right), WASP$^{\Delta CRIB}$ (*wasA*⁻/GFP-WASP$^{\Delta CRIB}$, bottom left), or WASP^{**CRIB} (*wasA*⁻/GFP-WASP^{**CRIB}, bottom right). Error bars represent SEM.

(F) Frequency of active Rac accumulation at the back of *wasA*⁻ cells and *wasA*⁻ cells expressing wild-type WASP or WASP CRIB mutants. *wasA*⁻ (n = 11 cells): 88.2% ± 3.5%; *wasA*⁻/GFP-WASP (n = 28 cells): 12.7% ± 1.0%; *wasA*⁻/GFP-WASP$^{\Delta CRIB}$ (n = 24 cells): 78.1% ± 2.0%; *wasA*⁻/GFP-WASP^{**CRIB} (n = 28 cells): 76.7% ± 2.2%; means ± SEM. One-way ANOVA; *wasA*⁻/GFP-WASP versus *wasA*⁻/GFP-WASP$^{\Delta CRIB}$: $p < 0.0001$; *wasA*⁻/GFP-WASP versus *wasA*⁻/GFP-WASP^{**CRIB}: $p < 0.0001$.

(G) Migratory speed of *wasA*⁻ cells and *wasA*⁻ cells expressing wild-type WASP or WASP CRIB mutants. *wasA*⁻ (n = 45 cells): 5.0 ± 0.1 μm/min; *wasA*⁻/GFP-WASP (n = 44 cells): 6.9 ± 0.2 μm/min; *wasA*⁻/GFP-WASP$^{\Delta CRIB}$ (n = 44 cells): 5.9 ± 0.2 μm/min; *wasA*⁻/GFP-WASP^{**CRIB} (n = 41 cells): 5.5 ± 0.1 μm/min; means ± SEM. Kruskal-Wallis test; *wasA*⁻/GFP-WASP versus *wasA*⁻/GFP-WASP$^{\Delta CRIB}$: $p = 0.0004$; *wasA*⁻/GFP-WASP versus *wasA*⁻/GFP-WASP^{**CRIB}: $p < 0.0001$.

considerations. It highlights our poor understanding of the uropod, which may simplistically be seen as a black hole where unnecessary migratory components are passively drawn, and it underscores our incomplete knowledge of how cells achieve spatial and functional segregation of WASP and SCAR/WAVE.

We propose a novel role for WASP as a homeostatic regulator of active Rac. We envision a model whereby WASP at CCPs, in addition to triggering Arp2/3-complex-mediated actin polymerization, also removes active Rac from the membrane through its CRIB motif. In wild-type cells, we cannot detect the fluorescent active Rac marker at endocytic *puncta*, primarily for two reasons. First, active Rac that is recruited to a CCP is WASP bound, and thus unable to also bind our active Rac marker; second, the high membrane background masks the few active Rac molecules that, we predict, are recruited by each CCP. WASP-mediated clearance of active Rac will obviously occur faster when GTPases are excessively activated, and slower when there is little GTPase activation, so cellular levels of active Rac are maintained. Spatially, given that CME occurs mostly at the rear of migrating cells, active Rac will be removed faster there. Thus, WASP will ensure that active Rac is removed from the cell rear, maintaining front-rear polarity.

Our proposed role of WASP does not exclude other models of cell polarity. Rather, WASP-mediated clearance of active Rac may collaborate with other mechanisms underpinning spatial confinement of active Rac, such as membrane tension [61], in maintaining a dominant front.

WASP and CRIB Motifs: "The Cowl Does Not Make the Monk"

When WASP was first discovered, the presence of a CRIB motif led researchers to conclude that its activity was dependent on GTPases [3]. This now seems an oversimplification. We have shown here that WASP does not rely on the availability of (nor on the interaction with) active Rac to recruit Arp2/3 complex, or drive actin polymerization, during CME. On the other hand, WASP depends on a direct, CRIB-mediated, interaction with

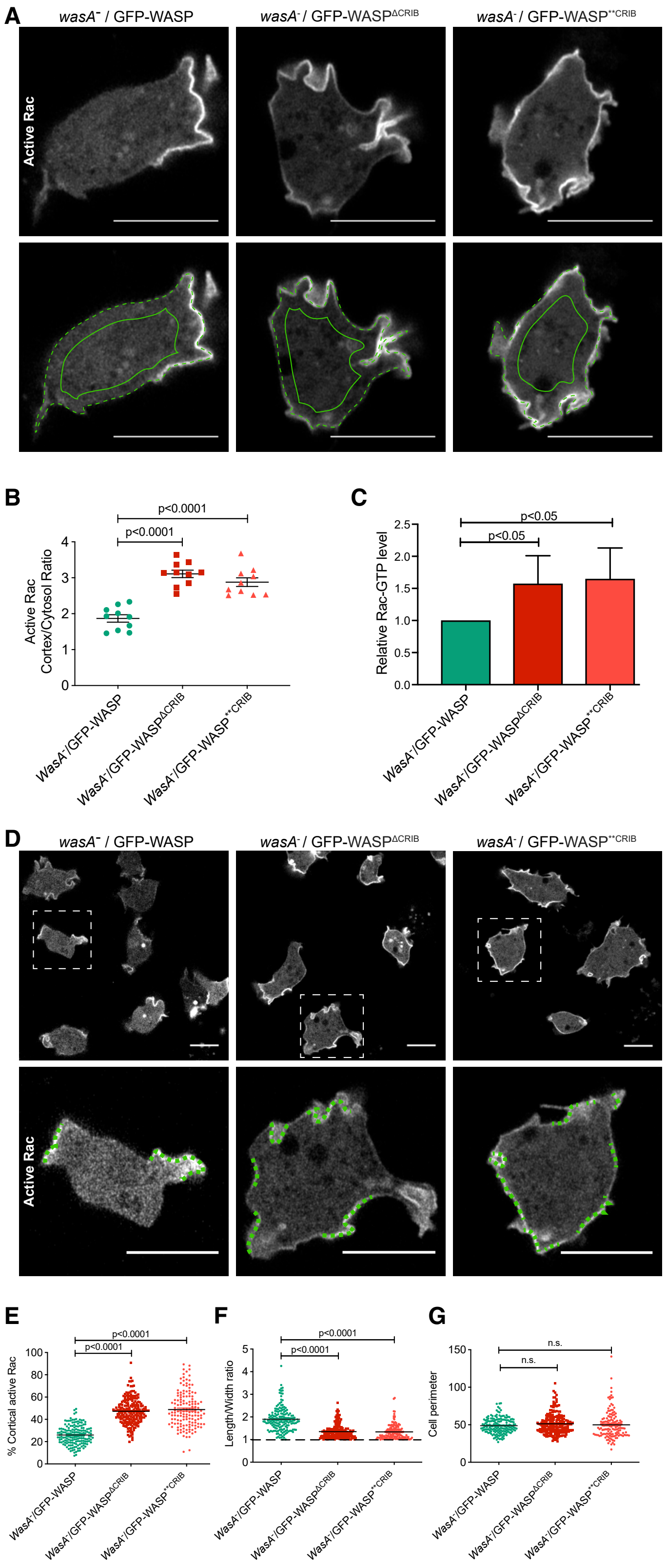

Figure 6. WASP Requires a Functional CRIB Motif to Maintain Homeostatic Levels of Active Rac

(A) Airyscan confocal imaging of *wasA*$^{-}$ cells co-expressing active Rac marker (PakB CRIB-mRFPmars2) and GFP-tagged WASP (wild-type, ΔCRIB, or **CRIB; green channel not shown). Images were acquired consistently through the mid-point of cells. The ratio between the intensity of the active Rac marker along the plasma membrane (dashed line) and the cytosol (solid line) was measured. Scale bars represent 10 μm.

(B) Quantification of active Rac marker membrane:cytosol intensity ratio from multiple cells as in (A) *wasA*$^{-}$/GFP-WASP (n = 10 cells): 1.9 ± 0.1; *wasA*$^{-}$/GFP-WASP$^{\Delta CRIB}$ (n = 10 cells): 3.1 ± 0.1; *wasA*$^{-}$/GFP-WASP^{**CRIB} (n = 10 cells): 2.9 ± 0.1; means ± SEM. One-way ANOVA; *wasA*$^{-}$/GFP-WASP versus *wasA*$^{-}$/GFP-WASP$^{\Delta CRIB}$: $p < 0.0001$; *wasA*$^{-}$/GFP-WASP versus *wasA*$^{-}$/GFP-WASP^{**CRIB}: $p < 0.0001$.

(C) Pull-down assay for active Rac levels. Active Rac was selectively precipitated from *wasA*$^{-}$ cells expressing wild-type or CRIB mutant WASPs, using GST-PAK-CRIB as bait. Active Rac was measured by western blot using an anti-Rac antibody and a fluorescent secondary antibody (n = 3 independent experiments; single-tailed t test).

(D) Airyscan confocal imaging of *wasA*$^{-}$ cells co-expressing active Rac marker and GFP-tagged WASP (wild-type, ΔCRIB, or **CRIB; green channel not shown). Cells expressing wild-type WASP (left) accumulate active Rac on a small proportion of their plasma membrane (dashed lines within inset). Cells expressing WASP$^{\Delta CRIB}$ or WASP^{**CRIB} (center and right, respectively) accumulate active Rac in a larger proportion of their plasma membrane (dashed lines within inset). Scale bars represent 10 μm.

(E) Percentage of plasma membrane enriched in active Rac in cells expressing wild-type or CRIB-mutated WASPs. Multiple cells from an experiment as in (C) *wasA*$^{-}$/GFP-WASP (n = 168 cells): 25.9% ± 0.7%; *wasA*$^{-}$/GFP-WASP$^{\Delta CRIB}$ (n = 204 cells): 47.3% ± 0.8%; *wasA*$^{-}$/GFP-WASP^{**CRIB} (n = 145 cells): 48.8% ± 1.3%; means ± SEM. Mann-Whitney test; *wasA*$^{-}$/GFP-WASP versus *wasA*$^{-}$/GFP-WASP$^{\Delta CRIB}$: $p < 0.0001$; *wasA*$^{-}$/GFP-WASP versus *wasA*$^{-}$/GFP-WASP^{**CRIB}: $p < 0.0001$. Related to Figure S4.

(F) Length-to-width ratio of cells expressing wild-type or CRIB-mutated WASP. Multiple cells from an experiment as in (C) *wasA*$^{-}$/GFP-WASP (n = 168 cells): 1.9 ± 0.04; *wasA*$^{-}$/GFP-WASP$^{\Delta CRIB}$ (n = 204 cells): 1.4 ± 0.02; *wasA*$^{-}$/GFP-WASP^{**CRIB} (n = 145 cells): 1.3 ± 0.03; means ± SEM. Mann-Whitney test; *wasA*$^{-}$/GFP-WASP versus *wasA*$^{-}$/GFP-WASP$^{\Delta CRIB}$: $p < 0.0001$; *wasA*$^{-}$/GFP-WASP versus *wasA*$^{-}$/GFP-WASP^{**CRIB}: $p < 0.0001$.

(G) Perimeter of cells expressing wild-type or CRIB-mutated WASP. Multiple cells from an experiment as in (C) *wasA*$^{-}$/GFP-WASP (n = 168 cells): 48.8 ± 0.8; *wasA*$^{-}$/GFP-WASP$^{\Delta CRIB}$ (n = 204 cells): 51.3 ± 0.9; *wasA*$^{-}$/GFP-WASP^{**CRIB} (n = 145 cells): 49.9 ± 1.5; means ± SEM. Kruskal-Wallis test; *wasA*$^{-}$/GFP-WASP versus *wasA*$^{-}$/GFP-WASP$^{\Delta CRIB}$: $p = 0.56$; *wasA*$^{-}$/GFP-WASP versus *wasA*$^{-}$/GFP-WASP^{**CRIB}: $p = 0.84$.

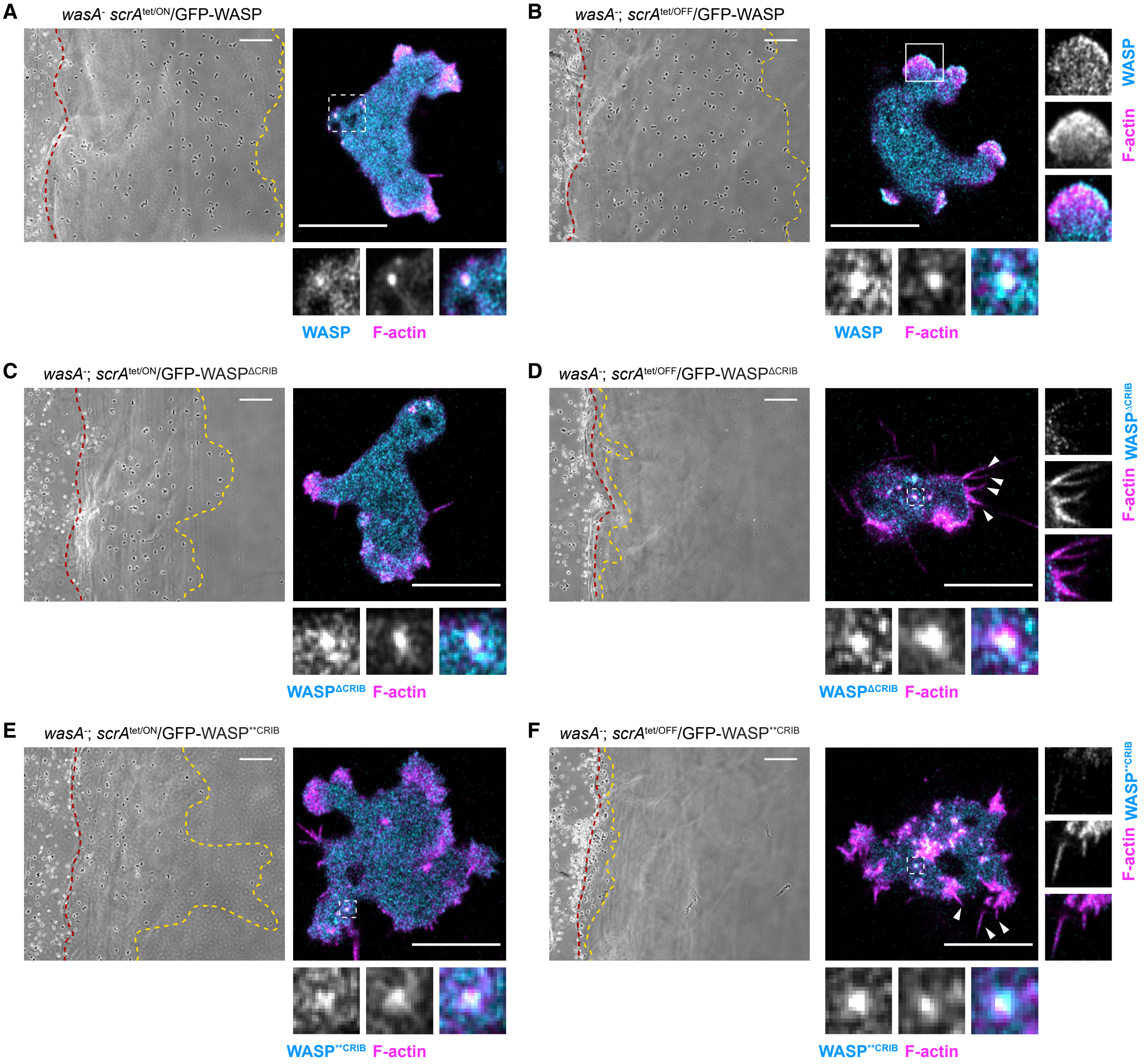

Figure 7. WASP Requires a Direct Interaction with Active Rac to Drive Pseudopod Extension in the Absence of SCAR/WAVE

(A) Inducible double null cells expressing GFP-WASP kept in the presence of tetracycline to maintain SCAR/WAVE expression (*wasA*$^{-}$ *scrA*$^{tet/ON}$/GFP-WASP) were allowed to chemotax and imaged by phase-contrast microscopy. Cells migrate efficiently from start (red line) to end (yellow line). Scale bar represents 100 μm. Live imaging (right-hand panel) shows GFP-WASP localizing to actin-rich *puncta* at the cell rear (square). Scale bar represents 10 μm.

(B) Inducible double-null cells expressing GFP-WASP kept in the absence of tetracycline to suppress SCAR/WAVE expression (*wasA*$^{-}$; *scrA*$^{tet/OFF}$/GFP-WASP) were allowed to chemotax and imaged by phase-contrast microscopy. Cells migrate efficiently from start (red line) to end (yellow line). Scale bar represents 100 μm. Live imaging (right-hand panel) shows GFP-WASP localizing to actin-rich *puncta* at the cell rear (dashed square) and at pseudopods generated at the front (solid square). Scale bar represents 10 μm.

(C) Inducible double-null cells expressing GFP-WASP$^{\Delta CRIB}$, kept in the presence of tetracycline to maintain SCAR/WAVE expression (*wasA*$^{-}$; *scrA*$^{tet/ON}$/GFP-WASP$^{\Delta CRIB}$), were allowed to chemotax and imaged by phase-contrast microscopy. Cells are able to migrate from start (red line) to end (yellow line), although less efficiently than cells shown in (A). Scale bars represent 100 μm. Live imaging (right-hand panel) shows GFP-WASP$^{\Delta CRIB}$ localizing at actin-rich *puncta* accumulated at the enlarged rear (squares). Scale bars represent 10 μm.

(D) Inducible double-null cells expressing GFP-WASP$^{\Delta CRIB}$, deprived of tetracycline to suppress SCAR/WAVE expression (*wasA*$^{-}$; *scrA*$^{tet/OFF}$/GFP-WASP$^{\Delta CRIB}$), were allowed to chemotax and imaged using phase-contrast microscopy. These cells do not migrate under agarose (proximity of the red and yellow lines, start and end point, respectively). Scale bars represent 100 μm. Live imaging (right-hand panels) reveals that cells expressing GFP-WASP$^{\Delta CRIB}$ WASP do not generate pseudopods and form spiky protrusions instead (arrowheads). The ability of GFP-WASP$^{\Delta CRIB}$ to generate actin-rich *puncta* is not affected (dashed squares). Scale bars represent 10 μm.

(legend continued on next page)

active Rac to drive pseudopod extension in the absence of SCAR/WAVE. Therefore, WASP's requirement for active Rac appears to be context dependent, not an absolute requirement as initially thought. In the broader context, this means that inhibiting Rac may not of itself compromise WASP functionality. WASP has many physiological roles (e.g., invadopodia formation), and its CRIB motif is likely to be important for a subset of other processes that we have not explored.

STAR★METHODS

Detailed methods are provided in the online version of this paper and include the following:

- KEY RESOURCES TABLE
- LEAD CONTACT AND MATERIALS AVAILABILITY
- EXPERIMENTAL MODEL AND SUBJECT DETAILS
  - Cell culture
  - Biochemistry
- METHOD DETAILS
  - Cell transfection
  - Protein purification
  - GST-Rac pull-down assay and immunoblotting
  - Active Rac pull-down assay
  - Microscopy
  - Chemotaxis assay
  - Rac inhibitor treatment
  - Induction of G12V Rac1 expression
  - Image processing
- QUANTIFICATION AND STATISTICAL ANALYSIS
- DATA AND CODE AVAILABILITY

SUPPLEMENTAL INFORMATION

Supplemental Information can be found online at https://doi.org/10.1016/j.cub.2019.10.036.

ACKNOWLEDGMENTS

We thank Margaret O'Prey and Beatson Advanced Imaging Resource (BAIR) staff for assistance with microscopy, Stephen Barratt for assistance with cloning of the CRIB mutants, and Douwe Veltman for providing plasmids. Funding was provided by Cancer Research UK grants A15672, A24450, and multidisciplinary grant A20017. Sequences and gene expression data were provided by Dictybase. We are very grateful to members of the Insall and Machesky Labs for helpful comments on the manuscript.

AUTHOR CONTRIBUTIONS

Conceptualization, C.A. and R.H.I.; Methodology, C.A. and S.I.; Investigation, C.A., P.A.T., A.J.D., and K.S.; Review and Editing, C.A., R.H.I., and L.M.M.; Visualization, C.A. and R.H.I.; Supervision, C.A. and R.H.I.

DECLARATION OF INTERESTS

The authors declare no competing interests.

Received: April 25, 2019
Revised: October 1, 2019
Accepted: October 18, 2019
Published: November 27, 2019

REFERENCES

1. Mullins, R.D., Heuser, J.A., and Pollard, T.D. (1998). The interaction of Arp2/3 complex with actin: nucleation, high affinity pointed end capping, and formation of branching networks of filaments. Proc. Natl. Acad. Sci. USA *95*, 6181–6186.
2. Derry, J.M., Ochs, H.D., and Francke, U. (1994). Isolation of a novel gene mutated in Wiskott-Aldrich syndrome. Cell *78*, 635–644.
3. Miki, H., Miura, K., and Takenawa, T. (1996). N-WASP, a novel actin-depolymerizing protein, regulates the cortical cytoskeletal rearrangement in a PIP2-dependent manner downstream of tyrosine kinases. EMBO J. *15*, 5326–5335.
4. Miki, H., Sasaki, T., Takai, Y., and Takenawa, T. (1998). Induction of filopodium formation by a WASP-related actin-depolymerizing protein N-WASP. Nature *391*, 93–96.
5. Veltman, D.M., and Insall, R.H. (2010). WASP family proteins: their evolution and its physiological implications. Mol. Biol. Cell *21*, 2880–2893.
6. Davidson, A.J., Amato, C., Thomason, P.A., and Insall, R.H. (2018). WASP family proteins and formins compete in pseudopod- and bleb-based migration. J. Cell Biol. *217*, 701–714.
7. Kochubey, O., Majumdar, A., and Klingauf, J. (2006). Imaging clathrin dynamics in Drosophila melanogaster hemocytes reveals a role for actin in vesicle fission. Traffic *7*, 1614–1627.
8. Madania, A., Dumoulin, P., Grava, S., Kitamoto, H., Schärer-Brodbeck, C., Soulard, A., Moreau, V., and Winsor, B. (1999). The Saccharomyces cerevisiae homologue of human Wiskott-Aldrich syndrome protein Las17p interacts with the Arp2/3 complex. Mol. Biol. Cell *10*, 3521–3538.
9. Merrifield, C.J., Qualmann, B., Kessels, M.M., and Almers, W. (2004). Neural Wiskott Aldrich Syndrome Protein (N-WASP) and the Arp2/3 complex are recruited to sites of clathrin-mediated endocytosis in cultured fibroblasts. Eur. J. Cell Biol. *83*, 13–18.
10. Evans, I.R., Ghai, P.A., Urbančič, V., Tan, K.L., and Wood, W. (2013). SCAR/WAVE-mediated processing of engulfed apoptotic corpses is essential for effective macrophage migration in Drosophila. Cell Death Differ. *20*, 709–720.
11. Kunda, P., Craig, G., Dominguez, V., and Baum, B. (2003). Abi, Sra1, and Kette control the stability and localization of SCAR/WAVE to regulate the formation of actin-based protrusions. Curr. Biol. *13*, 1867–1875.
12. Veltman, D.M., King, J.S., Machesky, L.M., and Insall, R.H. (2012). SCAR knockouts in Dictyostelium: WASP assumes SCAR's position and upstream regulators in pseudopods. J. Cell Biol. *198*, 501–508.
13. Weiner, O.D., Rentel, M.C., Ott, A., Brown, G.E., Jedrychowski, M., Yaffe, M.B., Gygi, S.P., Cantley, L.C., Bourne, H.R., and Kirschner, M.W. (2006). Hem-1 complexes are essential for Rac activation, actin polymerization, and myosin regulation during neutrophil chemotaxis. PLoS Biol. *4*, e38.

(E) Inducible double-null cells expressing GFP-WASP^{**CRIB}, kept in the presence of tetracycline to maintain SCAR/WAVE expression (*wasA*$^{-}$; *scrA*$^{tet/ON}$/GFP-WASP^{**CRIB}), were allowed to chemotax and imaged by phase-contrast microscopy. Cells are able to migrate from start (red line) to end (yellow line), although less efficiently than cells shown in (A). Scale bars represent 100 μm. Live imaging (right-hand panel) shows GFP-WASP^{**CRIB} localizing at actin-rich *puncta* accumulated at the enlarged rear (squares). Scale bars represent 10μm.
(F) Inducible double-null cells expressing GFP-WASP^{**CRIB}, deprived of tetracycline to suppress SCAR/WAVE expression (*wasA*$^{-}$; *scrA*$^{tet/OFF}$/GFP-WASP^{**CRIB}), were allowed to chemotax and imaged using phase-contrast microscopy. These cells do not migrate under agarose (proximity of the red and yellow lines, start and end point, respectively). Live imaging (right-hand panels) reveals that cells expressing a GFP-WASP^{**CRIB} do not generate pseudopods and form spiky protrusions instead (arrowheads). The ability of GFP-WASP^{**CRIB} to generate actin-rich *puncta* is not affected (dashed squares). Scale bars represent 10 μm.

14. Samaniego, R., Sánchez-Martín, L., Estecha, A., and Sánchez-Mateos, P. (2007). Rho/ROCK and myosin II control the polarized distribution of endocytic clathrin structures at the uropod of moving T lymphocytes. J. Cell Sci. *120*, 3534–3543.

15. Davis, B.H., Walter, R.J., Pearson, C.B., Becker, E.L., and Oliver, J.M. (1982). Membrane activity and topography of F-Met-Leu-Phe-Treated polymorphonuclear leukocytes. Acute and sustained responses to chemotactic peptide. Am. J. Pathol. *108*, 206–216.

16. Damer, C.K., and O'Halloran, T.J. (2000). Spatially regulated recruitment of clathrin to the plasma membrane during capping and cell translocation. Mol. Biol. Cell *11*, 2151–2159.

17. Zhu, Z., Chai, Y., Jiang, Y., Li, W., Hu, H., Li, W., Wu, J.W., Wang, Z.X., Huang, S., and Ou, G. (2016). Functional coordination of WAVE and WASP in C. elegans neuroblast migration. Dev. Cell *39*, 224–238.

18. Banjade, S., Wu, Q., Mittal, A., Peeples, W.B., Pappu, R.V., and Rosen, M.K. (2015). Conserved interdomain linker promotes phase separation of the multivalent adaptor protein Nck. Proc. Natl. Acad. Sci. USA *112*, E6426–E6435.

19. Pils, S., Kopp, K., Peterson, L., Delgado Tascón, J., Nyffenegger-Jann, N.J., and Hauck, C.R. (2012). The adaptor molecule Nck localizes the WAVE complex to promote actin polymerization during CEACAM3-mediated phagocytosis of bacteria. PLoS ONE *7*, e32808.

20. Chen, Z., Borek, D., Padrick, S.B., Gomez, T.S., Metlagel, Z., Ismail, A.M., Umetani, J., Billadeau, D.D., Otwinowski, Z., and Rosen, M.K. (2010). Structure and control of the actin regulatory WAVE complex. Nature *468*, 533–538.

21. Eden, S., Rohatgi, R., Podtelejnikov, A.V., Mann, M., and Kirschner, M.W. (2002). Mechanism of regulation of WAVE1-induced actin nucleation by Rac1 and Nck. Nature *418*, 790–793.

22. Rohn, J.L., Sims, D., Liu, T., Fedorova, M., Schöck, F., Dopie, J., Vartiainen, M.K., Kiger, A.A., Perrimon, N., and Baum, B. (2011). Comparative RNAi screening identifies a conserved core metazoan actinome by phenotype. J. Cell Biol. *194*, 789–805.

23. Burbelo, P.D., Drechsel, D., and Hall, A. (1995). A conserved binding motif defines numerous candidate target proteins for both Cdc42 and Rac GTPases. J. Biol. Chem. *270*, 29071–29074.

24. Pirone, D.M., Carter, D.E., and Burbelo, P.D. (2001). Evolutionary expansion of CRIB-containing Cdc42 effector proteins. Trends Genet. *17*, 370–373.

25. Rohatgi, R., Ma, L., Miki, H., Lopez, M., Kirchhausen, T., Takenawa, T., and Kirschner, M.W. (1999). The interaction between N-WASP and the Arp2/3 complex links Cdc42-dependent signals to actin assembly. Cell *97*, 221–231.

26. Tomasevic, N., Jia, Z., Russell, A., Fujii, T., Hartman, J.J., Clancy, S., Wang, M., Beraud, C., Wood, K.W., and Sakowicz, R. (2007). Differential regulation of WASP and N-WASP by Cdc42, Rac1, Nck, and PI(4,5)P2. Biochemistry *46*, 3494–3502.

27. Han, J.W., Leeper, L., Rivero, F., and Chung, C.Y. (2006). Role of RacC for the regulation of WASP and phosphatidylinositol 3-kinase during chemotaxis of Dictyostelium. J. Biol. Chem. *281*, 35224–35234.

28. Rivero, F., Dislich, H., Glöckner, G., and Noegel, A.A. (2001). The Dictyostelium discoideum family of Rho-related proteins. Nucleic Acids Res. *29*, 1068–1079.

29. Lebensohn, A.M., and Kirschner, M.W. (2009). Activation of the WAVE complex by coincident signals controls actin assembly. Mol. Cell *36*, 512–524.

30. de la Roche, M., Mahasneh, A., Lee, S.F., Rivero, F., and Côté, G.P. (2005). Cellular distribution and functions of wild-type and constitutively activated Dictyostelium PakB. Mol. Biol. Cell *16*, 238–247.

31. Kraynov, V.S., Chamberlain, C., Bokoch, G.M., Schwartz, M.A., Slabaugh, S., and Hahn, K.M. (2000). Localized Rac activation dynamics visualized in living cells. Science *290*, 333–337.

32. Weiner, O.D., Marganski, W.A., Wu, L.F., Altschuler, S.J., and Kirschner, M.W. (2007). An actin-based wave generator organizes cell motility. PLoS Biol. *5*, e221.

33. Xu, J., Wang, F., Van Keymeulen, A., Herzmark, P., Straight, A., Kelly, K., Takuwa, Y., Sugimoto, N., Mitchison, T., and Bourne, H.R. (2003). Divergent signals and cytoskeletal assemblies regulate self-organizing polarity in neutrophils. Cell *114*, 201–214.

34. Tal, T., Vaizel-Ohayon, D., and Schejter, E.D. (2002). Conserved interactions with cytoskeletal but not signaling elements are an essential aspect of Drosophila WASp function. Dev. Biol. *243*, 260–271.

35. Abdul-Manan, N., Aghazadeh, B., Liu, G.A., Majumdar, A., Ouerfelli, O., Siminovitch, K.A., and Rosen, M.K. (1999). Structure of Cdc42 in complex with the GTPase-binding domain of the 'Wiskott-Aldrich syndrome' protein. Nature *399*, 379–383.

36. Kim, A.S., Kakalis, L.T., Abdul-Manan, N., Liu, G.A., and Rosen, M.K. (2000). Autoinhibition and activation mechanisms of the Wiskott-Aldrich syndrome protein. Nature *404*, 151–158.

37. Merrifield, C.J., Feldman, M.E., Wan, L., and Almers, W. (2002). Imaging actin and dynamin recruitment during invagination of single clathrin-coated pits. Nat. Cell Biol. *4*, 691–698.

38. Aghamohammadzadeh, S., and Ayscough, K.R. (2009). Differential requirements for actin during yeast and mammalian endocytosis. Nat. Cell Biol. *11*, 1039–1042.

39. Boulant, S., Kural, C., Zeeh, J.C., Ubelmann, F., and Kirchhausen, T. (2011). Actin dynamics counteract membrane tension during clathrin-mediated endocytosis. Nat. Cell Biol. *13*, 1124–1131.

40. Kaksonen, M., Sun, Y., and Drubin, D.G. (2003). A pathway for association of receptors, adaptors, and actin during endocytic internalization. Cell *115*, 475–487.

41. Fujimoto, L.M., Roth, R., Heuser, J.E., and Schmid, S.L. (2000). Actin assembly plays a variable, but not obligatory role in receptor-mediated endocytosis in mammalian cells. Traffic *1*, 161–171.

42. Gottlieb, T.A., Ivanov, I.E., Adesnik, M., and Sabatini, D.D. (1993). Actin microfilaments play a critical role in endocytosis at the apical but not the basolateral surface of polarized epithelial cells. J. Cell Biol. *120*, 695–710.

43. Gao, Y., Dickerson, J.B., Guo, F., Zheng, J., and Zheng, Y. (2004). Rational design and characterization of a Rac GTPase-specific small molecule inhibitor. Proc. Natl. Acad. Sci. USA *101*, 7618–7623.

44. Onesto, C., Shutes, A., Picard, V., Schweighoffer, F., and Der, C.J. (2008). Characterization of EHT 1864, a novel small molecule inhibitor of Rac family small GTPases. Methods Enzymol. *439*, 111–129.

45. Shutes, A., Onesto, C., Picard, V., Leblond, B., Schweighoffer, F., and Der, C.J. (2007). Specificity and mechanism of action of EHT 1864, a novel small molecule inhibitor of Rac family small GTPases. J. Biol. Chem. *282*, 35666–35678.

46. Moissoglu, K., Slepchenko, B.M., Meller, N., Horwitz, A.F., and Schwartz, M.A. (2006). In vivo dynamics of Rac-membrane interactions. Mol. Biol. Cell *17*, 2770–2779.

47. Veltman, D.M., Lemieux, M.G., Knecht, D.A., and Insall, R.H. (2014). PIP_3-dependent macropinocytosis is incompatible with chemotaxis. J. Cell Biol. *204*, 497–505.

48. Carnell, M., Zech, T., Calaminus, S.D., Ura, S., Hagedorn, M., Johnston, S.A., May, R.C., Soldati, T., Machesky, L.M., and Insall, R.H. (2011). Actin polymerization driven by WASH causes V-ATPase retrieval and vesicle neutralization before exocytosis. J. Cell Biol. *193*, 831–839.

49. Derivery, E., Sousa, C., Gautier, J.J., Lombard, B., Loew, D., and Gautreau, A. (2009). The Arp2/3 activator WASH controls the fission of endosomes through a large multiprotein complex. Dev. Cell *17*, 712–723.

50. King, J.S., Veltman, D.M., Georgiou, M., Baum, B., and Insall, R.H. (2010). SCAR/WAVE is activated at mitosis and drives myosin-independent cytokinesis. J. Cell Sci. *123*, 2246–2255.

51. Burdyniuk, M., Callegari, A., Mori, M., Nédélec, F., and Lénárt, P. (2018). F-Actin nucleated on chromosomes coordinates their capture by microtubules in oocyte meiosis. J. Cell Biol. *217*, 2661–2674.

52. Caridi, C.P., D'Agostino, C., Ryu, T., Zapotoczny, G., Delabaere, L., Li, X., Khodaverdian, V.Y., Amaral, N., Lin, E., Rau, A.R., and Chiolo, I. (2018). Nuclear F-actin and myosins drive relocalization of heterochromatic breaks. Nature *559*, 54–60.

53. Pocha, S.M., and Cory, G.O. (2009). WAVE2 is regulated by multiple phosphorylation events within its VCA domain. Cell Motil. Cytoskeleton *66*, 36–47.

54. Carlier, M.F., Nioche, P., Broutin-L'Hermite, I., Boujemaa, R., Le Clainche, C., Egile, C., Garbay, C., Ducruix, A., Sansonetti, P., and Pantaloni, D. (2000). GRB2 links signaling to actin assembly by enhancing interaction of neural Wiskott-Aldrich syndrome protein (N-WASp) with actin-related protein (ARP2/3) complex. J. Biol. Chem. *275*, 21946–21952.

55. Papayannopoulos, V., Co, C., Prehoda, K.E., Snapper, S., Taunton, J., and Lim, W.A. (2005). A polybasic motif allows N-WASP to act as a sensor of PIP(2) density. Mol. Cell *17*, 181–191.

56. Prehoda, K.E., Scott, J.A., Mullins, R.D., and Lim, W.A. (2000). Integration of multiple signals through cooperative regulation of the N-WASP-Arp2/3 complex. Science *290*, 801–806.

57. Benesch, S., Lommel, S., Steffen, A., Stradal, T.E., Scaplehorn, N., Way, M., Wehland, J., and Rottner, K. (2002). Phosphatidylinositol 4,5-biphosphate (PIP2)-induced vesicle movement depends on N-WASP and involves Nck, WIP, and Grb2. J. Biol. Chem. *277*, 37771–37776.

58. Kessels, M.M., Schwintzer, L., Schlobinski, D., and Qualmann, B. (2011). Controlling actin cytoskeletal organization and dynamics during neuronal morphogenesis. Eur. J. Cell Biol. *90*, 926–933.

59. Prehoda, K.E., and Lim, W.A. (2002). How signaling proteins integrate multiple inputs: a comparison of N-WASP and Cdk2. Curr. Opin. Cell Biol. *14*, 149–154.

60. Sarmiento, C., Wang, W., Dovas, A., Yamaguchi, H., Sidani, M., El-Sibai, M., Desmarais, V., Holman, H.A., Kitchen, S., Backer, J.M., et al. (2008). WASP family members and formin proteins coordinate regulation of cell protrusions in carcinoma cells. J. Cell Biol. *180*, 1245–1260.

61. Houk, A.R., Jilkine, A., Mejean, C.O., Boltyanskiy, R., Dufresne, E.R., Angenent, S.B., Altschuler, S.J., Wu, L.F., and Weiner, O.D. (2012). Membrane tension maintains cell polarity by confining signals to the leading edge during neutrophil migration. Cell *148*, 175–188.

62. Laevsky, G., and Knecht, D.A. (2001). Under-agarose folate chemotaxis of Dictyostelium discoideum amoebae in permissive and mechanically inhibited conditions. Biotechniques *31*, 1140–1142, 1144, 1146–1149.

63. Schindelin, J., Arganda-Carreras, I., Frise, E., Kaynig, V., Longair, M., Pietzsch, T., Preibisch, S., Rueden, C., Saalfeld, S., Schmid, B., et al. (2012). Fiji: an open-source platform for biological-image analysis. Nat. Methods *9*, 676–682.

64. Meijering, E., Dzyubachyk, O., and Smal, I. (2012). Methods for cell and particle tracking. Methods Enzymol. *504*, 183–200.

STAR★METHODS

KEY RESOURCES TABLE

REAGENT or RESOURCE	SOURCE	IDENTIFIER
Antibodies		
Rat monoclonal [3H9] Anti-GFP	Chromotek	Cat# 3h9-100; RRID: AB_10773374
Mouse monoclonal [GST.B6] Anti-GST	Abcam	Cat# ab18183; RRID: AB_444305
Mouse monoclonal [23A8] Anti-Rac1	MERCK	Cat# 05-389; RRID: AB_309712
Chemicals, Peptides, and Recombinant Proteins		
Rac inhibitor	Tocris	No. #3872
Guanosine 5′-[β,γ-imido]triphosphate trisodium salt hydrate (GTPγs)	Sigma-Aldrich	No. #G0635
Critical Commercial Assays		
Active Rac pull-down assay	Cytoskeleton	No. #BK035
Experimental Models: Cell Lines		
AX3, *wasA*::bsr^R	Insall Lab [6]	AD7_1
AX3, *wasA*::bsr^R parent	Insall Lab [6]	AD7_6
JH8, *scrA*:pyr5-6, $^{tet\text{-}on}$*scrA*; G418^R, *wasA*::bsr^R	Insall Lab [6]	AD12_21
Oligonucleotides		
CATTCTCTGGATCCCATTCCAAATCTTTCTCTTCCGATGAA	This manuscript	oSB5
AGAGAAAGATTTGGAATGGGATCCAGAGAATGG TTTTGATA	This manuscript	oSB6
TGCGTTGGTTGGAGCTGATGCTTCCAAA TCTTTCTCTTCCGAT	This manuscript	oSB15
GCATCAGCTCCAACCAACGCAAAACATG AAAGTCATATTGGTTGG	This manuscript	oSB16
Recombinant DNA		
pakB CRIB-GFP/LifeAct-mRFP co-expression vector	This manuscript	pAD149
GFP-WASP/pakB CRIB-mRFPmars2 co-expression vector	This manuscript	pCA37
GFP-WASP$^{\Delta CRIB}$/pakB CRIB-mRFPmars2 co-expression vector	This manuscript	pCA44
GFP-WASP^{**CRIB}/pakB CRIB-mRFPmars2 co-expression vector	This manuscript	pCA46
GFP- WASP$^{\Delta CRIB}$/LifeAct-mRFPmars2 co-expression vector	This manuscript	pCA73
GFP-WASP^{**CRIB} /LifeAct-mRFPmars2 co-expression vector	This manuscript	pCA74
GFP- WASP/LifeAct-mRFPmars2 co-expression vector	This manuscript	pCA75
HSPC300-GFP/mRFPmars2-ArpC4 co-expression vector	Insall Lab [50]	pDM604
GFP-WASP/CLC-mRFPmars co-expression vector	Insall Lab [6]	pDM656
$^{Dox/on}$G12V Rac1 inducible expression vector	Insall Lab [12]	pDM987
GFP-WASP$^{\Delta CRIB}$ expression vector	This manuscript	pSBZ9
GFP-WASP^{**CRIB} expression vector	This manuscript	pSBZ13
GFP-WASP expression vector	This manuscript	pSBZ14
GFP- WASP$^{\Delta CRIB}$/mRFPmars2-ArpC4 co-expression vector	This manuscript	pSBZ16
GFP- WASP^{**CRIB}/mRFPmars2-ArpC4 co-expression vector	This manuscript	pSBZ18
GFP- WASP/mRFPmars2-ArpC4 co-expression vector	This manuscript	pSBZ19
GFP- WASP$^{\Delta CRIB}$/CLC-mRFPmars co-expression vector	This manuscript	pSBZ21
GFP- WASP^{**CRIB}/CLC-mRFPmars co-expression vector	This manuscript	pSBZ23
GST-Rac1A bacterial expressing vector	Prof. Arjan Kortholt, University of Groningen	pGEXRac1A
GST-RacC bacterial expression vector	Prof. Arjan Kortholt, University of Groningen	PGEXRacC

(*Continued on next page*)

Continued		
REAGENT or RESOURCE	SOURCE	IDENTIFIER
Software and Algorithms		
Fiji (image handling)	National Institutes of Health	https://fiji.sc
Prism (statistics)	GraphPad	https://www.graphpad.com/scientific-software/prism/

LEAD CONTACT AND MATERIALS AVAILABILITY

Further information and requests for reagents should be directed to and will be fulfilled by the Lead Contact, Clelia Amato (clelia.amato@ed.ac.uk). Recombinant DNA generated during this study has not been deposited in a public repository but is available from the Lead Contact on request.

EXPERIMENTAL MODEL AND SUBJECT DETAILS

Cell culture

Dictyostelium wild-type, *wasA* knockout, and inducible double null cells were grown at 22°C on Petri dishes in HL5 supplemented with vitamins and micro-elements (Formedium).

Biochemistry

E. coli BL21 cells transformed with bacterial expression vectors were plated on ampicillin-containing SM plates, or kept in LB medium plus ampicillin in shaking conditions (100 or 200 rpm) at room temperature or 37°C as needed.

METHOD DETAILS

Cell transfection

Prior to transfection, *Dictyostelium* cells were resuspended in E-buffer (10 mM $KNaPO_4$, pH 6.1, 50 mM sucrose), incubated with DNA of interest, and electroporated at 500V using the ECM399 system (Harvard Apparatus UK). Transfected cells were then transferred in Petri dishes containing medium, and selected 24 hour later by addition of 50 μg/ml hygromycin.

Protein purification

E. coli BL21 cells were transformed with a pGEXRac1A-encoding vector by heat-shock at 42°C, and plated overnight on ampicillin-containing SM plates. One colony was transferred to a tube containing LB medium (1% Bacto-tryptone, 0.5% Bacto-yeast extract, 17 mM NaCl, pH 7) plus ampicillin, and kept in shaking condition (200 rpm) at 37°C until it reached the OD_{600} of 2. The bacterial suspension was then transferred in a larger volume of LB medium plus ampicillin, and kept overnight in shaking conditions (100 rpm) at room temperature. Once the bacterial suspension reached the OD_{600} of 0.4, 500 μM IPTG (Isopropyl β-D-1 thiogalactopyranoside) was added to yield the expression of GST-Rac1A, and bacteria were kept overnight in shaking conditions (100 rpm) at room temperature. Bacteria were then spun down using the Avanti J6-MI centrifuge (Beckman Coulter) at 4°C 3000 rpm for 20 minutes, and lysed using a detergent-based lysis buffer (1% Triton X-100, Halt Protease Inhibitor Cocktail, 1mM DTT). The lysate was sonicated 10 times at the maximum power with 10 s interval between cycles using a Soniprep 150 (MSE), and spun down at 4°C for 10 minutes at 12096 × g using an Avanti J-25 centrifuge (Beckman Coulter). The sonicated lysate was incubated for 2 hours at 4°C with beads (Glutathione High Capacity Magnetic Agarose Beads, Sigma-Aldrich) previously washed in Rac buffer (50 mM Tris HCl pH 7.5, 100 mM NaCl, Halt Protease Inhibitor Cocktail, 1mM DTT). The beads were then washed in Rac buffer and incubated with 1 mM GTPγs (Guanosine 5′-[β,γ-imido]triphosphate trisodium salt hydrate, Sigma-Aldrich) overnight at 4°C in the presence of 15 mM EDTA and 100 units of CIP (Alkaline Phosphatase, Calf Intestinal, NEB). $MgCl_2$ was ultimately added to the tube at the final concentration of 60 mM in order to close the Rac's nucleotide-binding pocket.

GST-Rac pull-down assay and immunoblotting

Dictyostelium cells were resuspended in lysis buffer (50 mM Tris HCl pH 8, 100 mM NaCl, 30 mM $MgCl_2$, 0.1% Triton X-100, Halt Protease Inhibitor Cocktail, 1mM DTT) and the resulting lysate added to the tube containing GTPγs-Rac1A-loaded beads for 1 hour at 4°C. The tubes were placed on a magnet and washed twice in washing buffer (50 mM Tris HCl pH 8, 100 mM NaCl, 30 mM $MgCl_2$, Halt Protease Inhibitor Cocktail, 1mM DTT). Beads were then washed twice with 1 mL of washing buffer, resuspended in NuPAGE LDS Sample Buffer (ThermoFisher) and incubated at 100°C for 5 minutes. Proteins contained in the eluted fraction were separated using NuPAGE 4%–12% Bis-Tris protein gels (ThermoFisher). Protein were transferred on nitrocellulose membrane (Amersham Protran®, Merck) at 80V for 1 hour. The membrane was blocked for 1-hour in the presence of 5% semi-skimmed milk diluted in TBS. The blocked membrane was incubated overnight at 4°C with the desired primary antibody. The membrane

was washed three times with TBST and incubated with the appropriate secondary antibody diluted in 5% BSA in TBST. The membrane was washed three times with TBST, scanned using Odyssey CLx (LI-COR) and analyzed using Image Studio Lite^{TM} software (LI-COR).

Active Rac pull-down assay

Active Rac levels were determined by pull-down assay using GST-PAK-CRIB immobilised to Glutathione beads according to manufacturer's instructions (Cytoskeleton, Inc, Active Rac Kit). Briefly, *wasA*- cells expressing GFP-WASP (*wasA*-/GFP-WASP) or respective CRIB mutants (*wasA*-/GFP-WASP^{**CRIB} and *wasA*-/GFP-WASP$^{\Delta CRIB}$) were collected from a confluent 30 cm Petri dish and resuspended in lysis buffer supplemented with HALT^{TM} EDTA-free Protease Inhibitor Cocktail at 4°C. After a brief clarification at 10,000 × g for a minute, the supernatant is loaded into GST-PAK-CRIB beads in Eppendorf tubes and incubated for 1 hour at 4°C with gentle rotation. The washed beads were then resuspended in 2x sample buffer and boiled for 3 minutes at 95°C. Proteins contained in the eluted fractions were separated using NuPAGE 12% Bis-Tris protein gels (ThermoFisher) and transferred into nitrocellulose membrane as described previously. The membrane is then blocked for 30 minutes in the presence of 5% semi-skimmed milk diluted in TBS. The blocked membrane was incubated overnight at 4°C with a mouse monoclonal anti-Rac antibody (1:500 dilution). The membrane was washed once with TBST and incubated with appropriate secondary antibody diluted in 5% BSA in TBST. After three washes, the membrane was scanned using Odyssey CLx (LI-COR) and analyzed using Image Studio Lite^{TM} software (LI-COR). Wild-type cells expressing G12V Rac1A under a tetracycline-inducible promoter was used a positive control in the above experiments. In short, cells were treated with tetracycline to the final concentration of 10 μg/ml to induce Rac G12V expression. Cells were harvested 2-3 hours post-induction and processed as described above. The levels of active Rac is quantified using Image Studio Lite^{TM}.

Microscopy

Super-resolution microscopy was adopted to investigate the ability of WASP CRIB mutants to localize to clathrin-coated pits and to recruit the Arp2/3 complex. Images were acquires using a Zeiss LSM880 equipped with a 63x/1.40 NA objective. GFP was excited at 488 nm, RFP at 561 nm. Images were acquired using the ZEN imaging software every 1 or 2 s. TIRF microscopy was utilized to monitor the dynamics of clathrin, WASPs, Arp2/3 complex and actin on the ventral surface of the cells. Images were acquired using a modified Nikon Eclipse TE 2000-U microscope equipped with a photometrics Evolve 512 camera and a DualView DV2 emission splitter. GFP and RFP were excited using 473 nm and 561 nm wavelengths respectively. A 100x/1.40 NA TIRF objective was used. Images were acquired every 1 or 2 s using the MetaMorph software. Phase-contrast microscopy was performed to test the ability of cells expressing WASP CRIB mutants to migrate following loss of SCAR/WAVE. Images were acquired using a Nikon ECLIPSE TE2000-E inverted microscope with a 10x/0.30 NA objective and equipped with a QImaging Retiga EXi digital camera. Spinning disk confocal microscopy was utilized to monitor WASP dynamics upon expression of dominant active Rac. Images were acquired using a Nikon Ti-E inverted microscope equipped with a Yokogawa CSU-X spinning disc confocal unit in combination with a High resolution Andor Neo sCMOS camera. A 100x/1.4 NA objective was used. GFP was excited at 488 nm, RFP at 561 nm. Images were acquired using the Andor IQ 2 software.

Chemotaxis assay

The under-agarose chemotaxis assay [62] was used to investigate the ability of cells expressing no WASP or WASP CRIB mutants to confine active Rac at the leading edge during migration, as well as the ability of inducible double null cells expressing WASP CRIB mutants to generate pseudopods following loss of SCAR/WAVE. A 50mm glass bottom dish (MatTek) was first treated with 10 mg/ml BSA to reduce the resistance encountered by cells crawling under agarose. Seakem GTG agarose was dissolved to the final concentration of 0.4% in LoFlo (Formedium), a low fluorescence medium that improves the quality of imaging. Dissolved agarose was cast on a pre-treated glass bottom dish and allowed to set. Once set, the agarose was cut using a scalpel so to create two wells separated by a 5 mm bridge; this was gently wriggled loose to facilitate cells' crawling. Cells of interest were resuspended in LoFlo, counted using CASY® Model TT Cell Counter (Innovatis), and diluted to a final concentration of 5×10^5 cells/ml. 200 μL of cell suspension was seeded on the left well created within the agarose layer. The other well was filled with 100 μM folic acid diluted in LoFlo. A square coverslip was carefully lowered down in order to cover both wells and prevent evaporation; the glass bottom dish was thereafter incubated at 22°C. Imaging was performed 3-10 hours after setting up the assay.

Rac inhibitor treatment

Cells expressing a red-tagged active Rac marker (PakB CRIB-mRFPmars2) or a GFP-tagged WASP alongside re-tagged LifeAct or Arp2/3 complex were resuspended in LoFlo, and 10^5 cells seeded on a borosilicate glass 8 well chamber (ThermoFisher). Cells were allowed to adhere and then treated with the Rac inhibitor EHT1864. Images were acquired prior to addition of the inhibitor (to monitor the dynamics of the probe in the presence of active Rac), during addition of the drug (to visualize its most acute effects) and for 10-12 minutes after treatment (to verify whether cells could recover).

Induction of G12V Rac1 expression

Cells expressing G12V Rac1A under a tetracycline-inducible promoter along with GFP-WASP or RFP-tagged active Rac marker were resuspended in LoFlo and seeded to a final concentration of 5×10^5 cells/ml on a borosilicate glass 8 well chamber (ThermoFisher) or

on a 35 mm glass bottom dish (MatTek). Once adhering, cells were treated with tetracycline to the final concentration of 10 μg/ml to yield G12V Rac1A expression. Cells were images from the moment of tetracycline addition up to 2-3 hours post-induction.

Image processing

Images acquired using phase-contrast, TIRF or confocal microscopes were exported as. TIFF files, while those acquired using the super-resolution confocal were exported as. CZI files. Images were all imported into Fiji software [63] and processed as required, including cropping and linear brightness/contrast adjustment. TIRF images were processed using the Windowed-Sinc Filter (Kunito Yoshida, Department of Biological Sciences, Imperial College London). Clathrin, WASP and Arp2/3 complex *puncta* dynamics was monitored using the MTrackJ plugin [64]. The same plugin was utilized along with a Chemotaxis Tool plugin (Gerhard Trapp and Elias Horn, ibidi GmbH) to measure speed and directionality of migrating cells. Images acquired using the super-resolution confocal microscope were subjected to Airyscan processing and to a 2D automatic deconvolution.

QUANTIFICATION AND STATISTICAL ANALYSIS

Unpaired t test, Mann-Whitney test, one-way ANOVA with a Kruskal-Wallis multiple comparison test or a Tukey's multiple comparisons test were performed to generate p values and test for statistical significance using GraphPad Prism 7 or 8. Plots were generated using the same software. Statistical details of experiments, including n, mean, SEM and p values, are indicated in figure legends.

DATA AND CODE AVAILABILITY

This study did not generate any unique datasets or code.

Current Biology, Volume *29*

Supplemental Information

WASP Restricts Active Rac to Maintain Cells' Front-Rear Polarization

Clelia Amato, Peter A. Thomason, Andrew J. Davidson, Karthic Swaminathan, Shehab Ismail, Laura M. Machesky, and Robert H. Insall

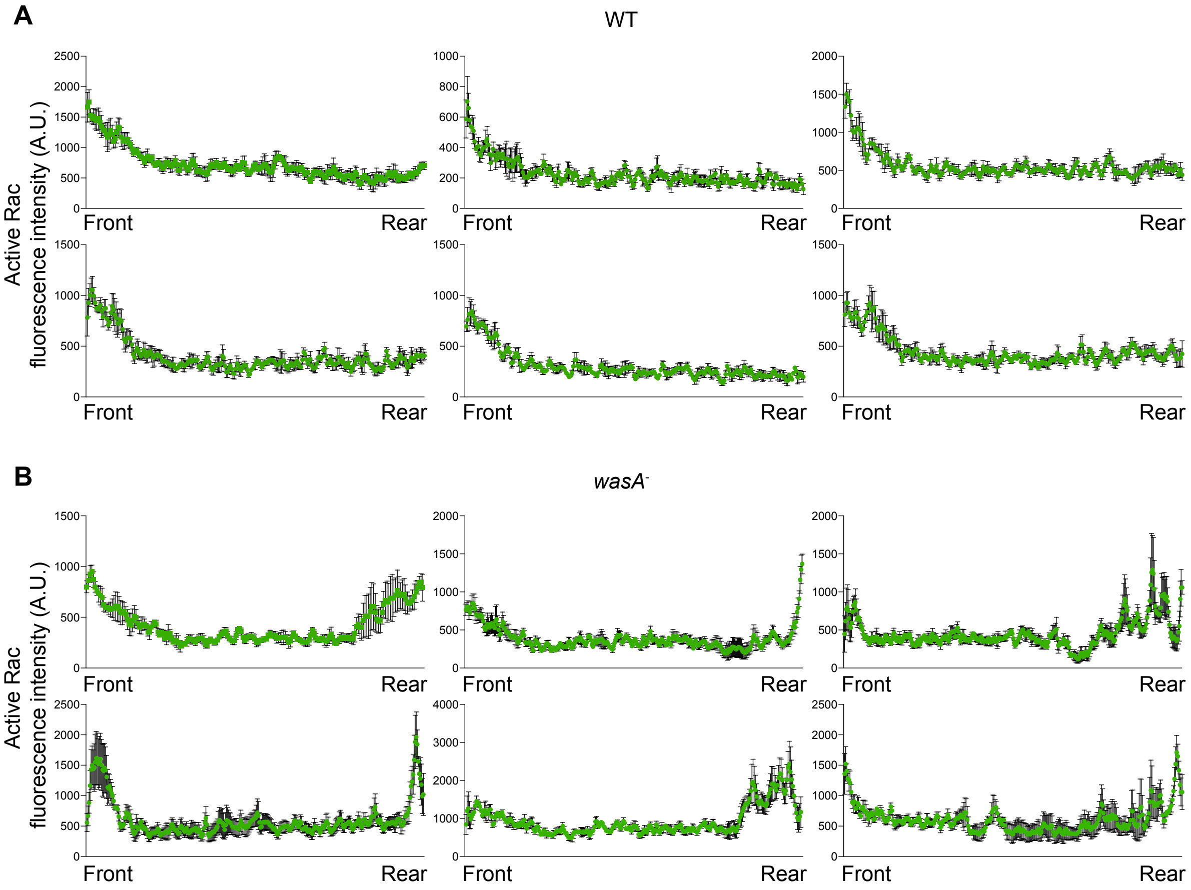
A
WT
Active Rac fluorescence intensity (A.U.)
Front
Rear
B
wasA-
Active Rac fluorescence intensity (A.U.)
Front
Rear

Figure S1. *wasA*⁻ cells accumulate active Rac at the rear. Related to Figure 1.

Each plot reports the fluorescence intensity of active Rac marker (PakB CRIB-GFP) along a line connecting front to rear of six representative wild type (WT) (A) and *wasA*⁻ (B) cells at four time-points. Active Rac is accumulated exclusively at the front in WT cells, but both at front and rear in *wasA*⁻ cells.

A

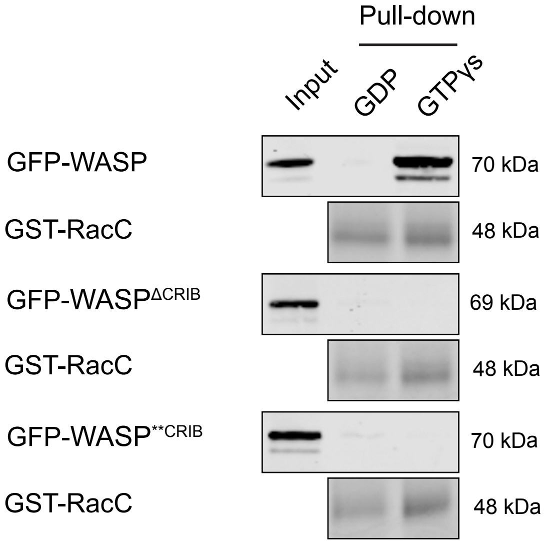
Pull-down
Input
GDP
GTPγs
GFP-WASP
70 kDa
GST-RacC
48 kDa
GFP-WASP^ΔCRIB
69 kDa
GST-RacC
48 kDa
GFP-WASP**CRIB
70 kDa
GST-RacC
48 kDa

Figure S2. Mutations in the WASP CRIB motif abrogate binding to active RacC. Related to Figure 2.

(A) GFP-WASP (first panel) interacts with active (GTPγs-bound) RacC, but not with inactive (GDP-bound) RacC. GFP- WASP$^{\Delta CRIB}$ and GFP-WASP^{**CRIB} (third and fifth panels) do not bind RacC, irrespective of its activation state (IB= anti-GFP). Anti-GST immunoblot was performed (second, fourth and sixth panels) to verify the expression of GST-RacC.

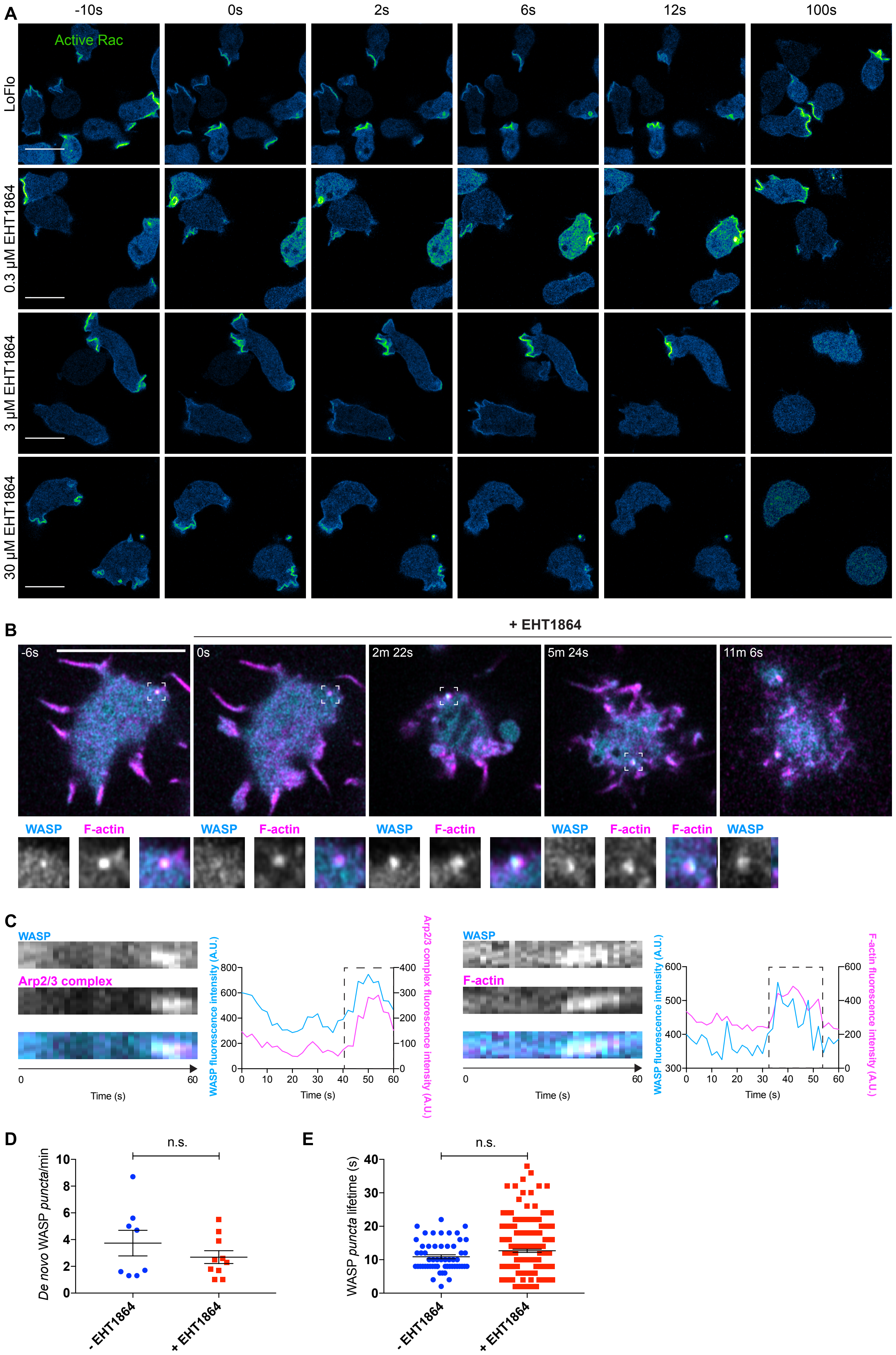

A
-10s
0s
2s
6s
12s
100s
Active Rac
LoFlo
0.3 µM EHT1864
3 µM EHT1864
30 µM EHT1864
B
+ EHT1864
-6s
0s
2m 22s
5m 24s
11m 6s
WASP
F-actin
C
WASP
Arp2/3 complex
Time (s)
WASP fluorescence intensity (A.U.)
Arp2/3 complex fluorescence intensity (A.U.)
F-actin
F-actin fluorescence intensity (A.U.)
D
n.s.
De novo WASP puncta/min
- EHT1864
+ EHT1864
E
WASP puncta lifetime (s)

Figure S3. Rac inhibition does not alter WASP dynamics nor its ability to trigger actin polymerisation on *puncta*. Related to Figure 4.

(A) Wild type cells expressing active Rac marker (PakB CRIB-mRFPmars2) were imaged prior to and upon addition of increasing concentration of the Rac inhibitor EHT1864. Before treatment (t=-10s), the active Rac marker is enriched on the plasma membrane, mostly at forming macropinosomes. Addition (t=0s) of low doses (0.3 μM, second line) of the inhibitor does not affect cell morphology nor the distribution of the active Rac marker. Addition (t=0s) of a higher dose (3 μM, third line) of the compound triggers a change in cell shape as well as a reduction of membrane-targeted active Rac marker. Further increase of the drug concentration (30 μM, bottom line) leads to a quicker and more dramatic effect. Scale bars=10 μm. As a control, cells were imaged prior to and upon addition of LoFlo, a low-fluorescence medium here utilised as buffer for the Rac inhibitor. As expected, adding LoFlo does not affect localisation of the active Rac marker, nor cell morphology (top line). (B) WASP's ability to generate F-actin-rich *puncta* upon chemical inhibition of Rac was investigated using Airyscan confocal imaging of *wasA*$^{-}$ cells expressing GFP-WASP and LifeAct-mRFPmars2. Before addition of the drug (t=-6s), WASP can be detected on individual spots (dashed square). Shortly after treatment (t=0s) with a high dose of the drug (50 μM), cells undergo a morphological change. As indicated by dashed squares (highlighted within insets) at given time-points, WASP is able to generate F-actin-rich *puncta* even after 11 minutes from the treatment. Scale bar=10 μm. (C) Left-hand side kymograph and fluorescence plot show a representative example of WASP generating an Arp2/3 complex-positive *punctum* in the presence of Rac inhibitor. Right-hand side kymograph and fluorescence plot report a representative example of WASP generating an F-actin-positive *punctum* in the presence of

Rac inhibitor. Both kymographs were obtained from a time-lapse as shown in B. (D) No significant difference can be measured between the number of WASP *puncta* generated per minute prior to and upon Rac inhibition. Untreated cells (-EHT1864, n=8 cells): 3.7 ± 1.0; treated cells (+EHT1864, n=10 cells): 2.7 ± 0.5; means ± SEM. Unpaired t-test, p=0.3130). (E) No significant difference can be measured between the lifetime of WASP *puncta* generated prior to and upon addition of the Rac inhibitor. Untreated cells (-EHT1864, n=57 *puncta*): 10.88s ± 0.59; treated cells (+EHT1864, n=213 *puncta*): 12.67s ± 0.49; means ± SEM. Mann-Whitney test, p=0.2023.

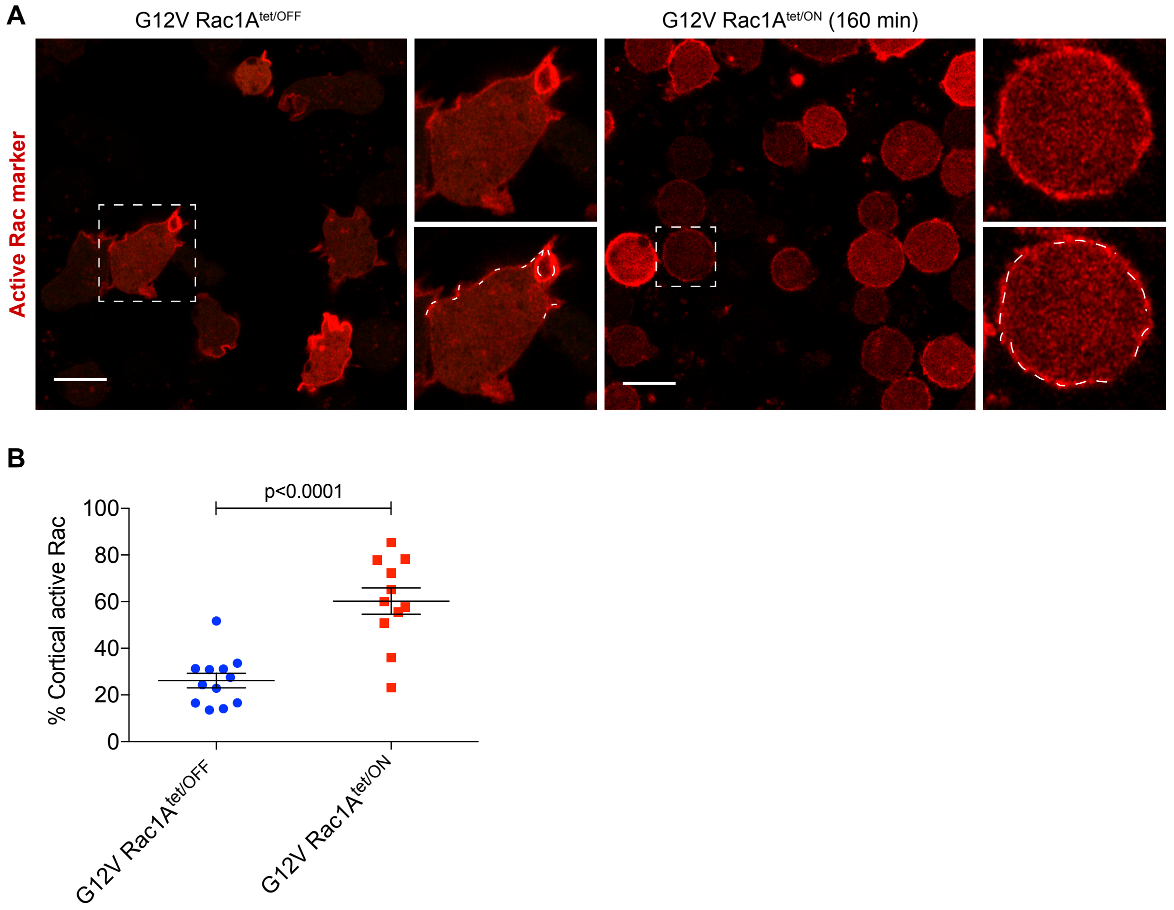
A
G12V Rac1A tet/OFF
G12V Rac1A tet/ON (160 min)
Active Rac marker
B
p<0.0001
% Cortical active Rac
100
80
60
40
20
0
G12V Rac1A tet/OFF
G12V Rac1A tet/ON

Figure S4. The proportion of plasma membrane labelled by the active Rac marker correlates with active Rac levels. Related to Figure 6.

(A) *wasA*$^{-}$ cells expressing GFP-WASP (rescue, green channel not shown), active Rac marker (pakB CRIB-mRFPmars2), and a tetracycline-inducible dominant active (G12V) Rac1A were imaged prior to and upon addition of tetracycline, and the percentage of plasma membrane labelled by the active Rac marker was measured. Prior to tetracycline treatment (left panels, G12V Rac1A$^{tet/OFF}$), the active Rac marker labels a small proportion of the plasma membrane. Upon addition of tetracycline (right panels, G12V Rac1A$^{tet/ON}$), cells accumulate the active Rac marker on a significantly larger proportion of their plasma membrane. (B) Quantification of the percentage (%) of plasma membrane labelled by the active Rac marker prior to and upon addition of tetracycline. - tetracycline (G12V Rac1A$^{tet/OFF}$, n=12 cells): 26.18% ± 3.12; +tetracycline (G12V Rac1A$^{tet/ON}$, n=11 cells): 60.25% ± 5.65; means ± SEM. Unpaired t-test, $p<0.0001$. Scale bars=10 μm.
